# Sexual function in chronic illness and cancer perspectives of the patient, partner, and healthcare provider; innovations; and updates: recommendations from the Fifth International Consultation on Sexual Medicine (ICSM 2024)

**DOI:** 10.1093/sxmrev/qeag002

**Published:** 2026-02-23

**Authors:** Kevan Richard Wylie, Michael G Kirby, Filippo Maria Nimbi, Michael Krychman, Jennifer Barsky Reese, Deborah C Marshall

**Affiliations:** Retired; Department of Neurosciences, University of Sheffield, S10 2TN, United Kingdom; British Society of Sexual Medicine, WS14 9JL, United Kingdom; Department of Theoretical and Applied Sciences (DiSTA), eCampus University, Via Isimbardi 10 - 22060 Novedrate, Italy; University of California Irvine, Department OB/GYN, Division of Gynecology, Oncology, Irvine, CA, United States; Southern California Center for Sexual Health and Survivorship Medicine, Newport Beach, CA, United States; Fox Chase Cancer Center, Cancer Prevention and Control Program, Philadelphia 19111 PA, United States; Icahn School of Medicine at Mount Sinai, New York, NY, United States

**Keywords:** diabetes mellitus, coronary vascular disease, chronic pain, breast cancer, gynecological cancer, oncology, mental health

## Abstract

**Introduction:**

Disorders of chronic illness and certain cancers are common and can pose a substantial burden to the individual and their sexual partners.

**Objectives:**

This manuscript reviews the substantial impact of chronic illness and cancer on sexual function from the perspectives of patients, partners, and healthcare providers. Convened by the Fifth International Consultation on Sexual Medicine, a multidisciplinary panel conducted a narrative review of current literature and expert consensus. The findings highlight both the direct and indirect effects of chronic diseases, breast and gynecological cancers, and oncological treatments on sexual health and relationships, emphasizing the importance of recognizing and addressing these issues to optimize care for individuals and their partners.

**Methods:**

Narrative review of the existing literature and consensus recommendations from the expert panel.

**Results:**

The direct and indirect impact of chronic disease and cancers and the impact of associated treatment interventions on sexual function and relationships is considerable.

**Conclusions:**

An understanding of the impact of chronic illness and cancers, and the related treatment of such can aid clinicians and researchers in providing optimal care to the individual and their partner(s).

## RECOMMENDATIONS

**SECTION A**

- The complications of DM represent a major health problem, macroangiopathy, microangiopathy, and neuropathy all leading to sexual dysfunction in both men and women. Strong.
- Risk factors, physical inactivity, obesity, metabolic syndrome, diabetes, and cardiovascular disease lead to low sexual desire and sexual dysfunction. Strong.
- Sexual problems may be a warning sign of underlying disease or a consequence of chronic diseases. Strong.
- Erectile dysfunction is a hallmark of CVD. Strong.
- Men with priapism are at increased risk for cardiovascular and cerebrovascular events in the years following a priapism. Weak
- Postmenopausal women, particularly those in the middle-age range, should be assessed for CV risk factors and FSD, sexual dysfunction is a harbinger of cardiovascular disease in postmenopausal women. Strong.
- Low sexual desire and sexual dysfunction are often associated with low testosterone levels and higher fat mass. Therefore, the prevalence of sexual problems in healthy middle-aged individuals may be associated with the presence of a portfolio of these risk factors. Strong.
- DM has a negative impact on male fertility, particularly the quality of sperm, semen constituents, sperm motility, and sperm DNA damage. Strong
- Ejaculatory dysfunction encompasses several disorders related to DM and its complications, such as premature ejaculation, anejaculation (AE), delayed ejaculation, retrograde ejaculation (RE), ejaculatory pain, anesthetic ejaculation, decreased ejaculate volume, and decreased force of ejaculation. The problems linked to ejaculatory dysfunction may lead to mediocre quality of life as both AE and RE are alleged to alter the fertility potential. Strong.
- The initial management for PE is controlling the patient’s blood glucose, in some cases this may allow recovery of the normal ejaculatory function. Amelioration of glycemic variability will improve PE in type 1 diabetes patients. In cases of concomitant ED and PE, ED should receive phosphodiesterase type 5 inhibitors before, or at least at the same time as other treatments. Strong.
- SD is commonly encountered in older T2DM patients and diabetic kidney disease (DKD) affects almost 50%. The eGFR has been significantly associated with SD, ED, and FSD, while SD and ED were proven to be significant determinants for the eGFR levels. Strong.
- Female sexual dysfunction is more common among women with type 1 diabetes than among women without type 1 diabetes. Patients with type 1 diabetes should be questioned in terms of sexual health. Health professionals should give more attention to and provide guidance regarding sexual function in both men and women with type 1 diabetes. Strong.
- S-Klotho plasma levels have been associated with sexual desire and sexual function in men and, also, with dyadic sexual desire and sexual function in women. S-Klotho plasma levels may represent a potential new biomarker for sexual desire and sexual function. Weak
- Testosterone therapy enhances insulin sensitivity in obese men with hypogonadism by decreasing fat mass, increasing lean mass, decreasing free fatty acids, and suppressing inflammation. Leading to, improved glycaemia, endothelial function, and lipid profile, together with improvement in the symptoms of hypogonadism, and potentially reducing cardiovascular risk. Strong.
- Testosterone therapy prevents progression from prediabetes to diabetes and improves HbA1c. Strong.
- Obesity is associated with hypogonadism, sexual dysfunction, and impaired fertility in men. Strong.
- Exercise protects against erectile dysfunction in men and sexual dysfunction in women and in addition improves sexual desire and sexual function in women. Strong.
- Bariatric surgery improves male and female sexual health. It is associated with significant increase in IIEF score and serum testosterone levels. Similar benefits in sexual function and self-esteem are seen in women. Strong.
- The role of different anti-diabetes therapies in the potential remission of ED and FSD, need to be explored, highlighting specific pathways whose activation or inhibition could be fundamental for sexual care in a diabetes setting. Strong.
- The Mediterranean diet is effective for the protection of sexual function. Furthermore, anti-hyperglycemic drugs seem to show an overall protective role on sexual function. Strong.
- Despite being used for ED, PDE5is have favorable effects on the cardiovascular system. Strong.
**SECTION B**
- Evaluating sexual functioning in CP condition is strongly suggested within the diagnostic or routine care since data report a high prevalence of sexual impairment in both women and men. Strong.
- In the evaluation, a multifactorial cause involving the CP condition per se, associated symptoms, treatments used, and relational and social experience of the condition should be considered. Strong.
- It is suggested to incorporate questionnaires considering sexual health in the CP assessment. Strong recommendation.
- Opioid-based therapy side effects, although not related to sexuality, can exacerbate sexual difficulties in CP patients. Strong.
- Using medications to address sexual dysfunction among opioid users, although commonly reported, should be evaluated case by case. Weak recommendation.
- It remains challenging to determine the extent to which antidepressant therapies directly impact sexuality versus inherent features of CP. Weak recommendation.
- Using the ReConnect model in both individual and group sessions may aid HCPs in supporting their patients’ sexual activity goals. Weak.
- Effective communication and the exploration of alternative forms of intimacy may facilitate personal empowerment, enabling individuals and couples to adapt and discover new strategies to sustain their sexual lives despite experiencing pain.
- Emphasis should be on mitigating symptoms associated with CP and promoting sexual satisfaction rather than solely concentrating on functioning. Weak.
**SECTION C**
- Vaginal moisturizers and lubricants should be considered frontline for the treatment of GSM in BCP (Strong)
- Other non-hormonal interventions (pharmacological/psychological): should be incorporated the treatment paradigm (Strong)
- Minimally absorbed local vaginal estradiol products maybe be used for refractory cases to OTC vaginal moisturizers and lubricants (Strong)
- Ospemifene and intravaginal DHEA can be considered for use in the BCP with GSM (Strong)
- CO2 Laser and Radiofrequency both show excellent promise for the treatment of GSM in BCP. However long term, sham controlled RCT is lacking, and the HCP should proceed with caution. (Strong)
- The HCP should be cautious of cash-based center’s or medi-spas which strongly advocate device-based interventions for breast cancer patients. (Strong)
  There is insufficient safety and efficacy data at the current time to recommend Testosterone use in breast cancer patients (Strong)
- Shared decision making should take place for the treatment of GSM in BC, P, and involve the patient, survivorship specialist and the oncological team (Strong).
**SECTION D**
- Pelvic floor function interventions, including pelvic floor training along with the use of dilators and other strategies, are well-tested (Strong).
- Vaginal dilators alone are promising but have a smaller evidence base (Strong).
- DHEA lacks rigorous testing in women with gynecologic cancer (Weak)
- Bupropion for improving sexual desire lacks substantial rigorous testing in women with gynecologic cancer (Weak).
- Ospemifene, an estrogen receptor agonist/antagonist, also known as a selective estrogen receptor modulator (SERM), has some promising evidence but lacks testing in large randomized controlled trials (Weak).
- Microablative fractional CO2 laser lack data is available in gynecologic cancer patients (Weak).
- Psychoeducational and behavioral interventions show benefit for women with gynecologic cancer in improving sexual function and these interventions have the benefit of few risks or negative side effects (Strong).
- Limited data exist showing that exercise interventions improve sexual function in endometrial cancer survivors, but they do not have side effects and can have other health benefits (Strong).
**SECTION E**
- Sexual function sparing approaches to definitive cancer treatment, such as those that involve functional anatomy sparing or tumor down staging, should be considered, when medically appropriate (Weak).
- When available, advanced imaging guidance should be utilized to minimize adverse effects of definitive local cancer treatments on sexual functioning (Weak).
- Shared-decision making can guide treatment choice when sexual function may be impacted (Weak).
- Equitable approaches to preventing sexual dysfunction due to oncologic treatments in female and sexual/gender minority persons are needed (Strong).

## Introduction

The direct and indirect impact of chronic disease, breast and gynecological cancers, and oncologic interventions more generally and the impact of associated treatment interventions on sexual function and relationships is considerable. These have been reported in previous consultations.^1–5^ This review addresses updates and innovations since the last consultation reported in 2015 specifically of chronic cardiovascular disease (CVD) states, diabetes mellitus (DM) and obesity, chronic pain (CP) conditions, breast and gynecological cancers, and oncologic interventions.

The consensus of a diverse group of global experts chose pertinent and clinically relevant conditions to review as a committee following extensive discussion and under the guidance and support of the task force of the International Consultation on Sexual Medicine (ICSM).

It is recognized that several common chronic conditions have not been discussed in this chapter, but we acknowledge existing reviews in the literature covering these topics such as colitis^6^ and dermatological conditions^7^ as well as general disabilities from chronic illness and disease.^8^^,^^9^ Whilst prostate cancer and some other chronic conditions are considered in more detail elsewhere within the consultation including, for instance, some neurological conditions,^10^ the general impact of ageing^11^ and the genitourinary syndrome of menopause,^12^ there will be some common recommendations that relate to people and their partners who are impacted by most chronic disease conditions and cancer.

The review is presented in five sections starting with an overview of CVD, DM, and obesity on sexual function, followed by CP conditions. The review then deals with breast cancer (BC), gynecological cancer, and then oncological interventions more generally.

## Methods

Under the auspices of the ICSM, a multidisciplinary panel of experts on disorders of sexual function in chronic illness and cancer, selected from geographically representative parts of the world, was tasked with providing evidence-based recommendations for management of sexual function from perspectives of the Patient, Partner, and Healthcare Provider. The panel met virtually by Zoom and in person at the ICSM meeting in Madrid, 2024.

The panel reviewed the global literature on sexual function in various chronic illnesses and cancers. Each individual member was tasked with researching discrete topics and performed their own review and data synthesis for their assignment. Draft recommendations were produced by each committee member and presented to the larger group for discussion and debate and agreement of the related recommendations from each section. All authors approved the final manuscript.

Following the ICSM guidance, all recommendations include assessment of the quality of evidence and assignment to recommendations as either strong or weak, based on the GRADE system.

## Results

Chronic disease conditions such as CVD, obesity and diabetes, CP, and breast and gynecological cancer states exert both direct and indirect effects on the body, with consequences that are multifaceted and frequently interactional. These impacts create a complex insult affecting several major systems, including the cardiovascular, neurological, endocrinological, and soft tissue structures.

Management of chronic diseases often involves a variety of interventions, ranging from pharmacological and surgical approaches to external treatments such as radiation and laser therapy. These interventions, while essential for disease management, can also contribute to a complex environment that may result in sexual dysfunction (SD).

SDs resulting from chronic disease and its treatment has a significant impact not only on affected individuals but also on their partners. This underscores the importance of recognizing the broader psychosocial effects of chronic disease management.

Given the considerable impact of SD related to chronic disease, the role of physicians and clinical care providers becomes especially important. Healthcare professionals (HCPs) are responsible for addressing these concerns, providing appropriate guidance, and offering relevant support and interventions to help patients and their partners navigate these challenges.

We discuss these common chronic disease states and cancers in the following section. We reiterate that while some findings that are reported are disease specific, there will be some common recommendations that relate to people and their partners who are impacted by most chronic disease conditions and cancer.

### Section A: Chronic CVD and obesity

#### Introduction

Sexual desire and sexual function are important components of quality of life, being intricately linked with overall health and relationship satisfaction. Numerous studies suggest that risk factors, physical inactivity, obesity, metabolic syndrome, diabetes, and CVD lead to low sexual desire and SD.

Sexual problems may be a warning sign of underlying disease or a consequence of chronic diseases. Low sexual desire and SD are often associated with low testosterone levels and higher fat mass. Therefore, the prevalence of sexual problems in healthy middle-aged individuals may be associated with the presence of a portfolio of these risk factors. This review looks at the key papers studying this link over the last 5 years.

#### Diabetes mellitus

A chronic metabolic disease characterized by elevated levels of blood glucose, is among the most common chronic diseases. The incidence and prevalence of DM has been increasing over the years. The complications of DM represent a major health problem, macroangiopathy, microangiopathy, and neuropathy all leading to SD in both men and women.^14–20^

Many studies have shown that DM has a negative impact on male fertility, particularly the quality of sperm, semen constituents, sperm motility, and sperm DNA damage. It is possible that this may not only result in subfertility or infertility but will also pass to the offspring and cause subfertility or infertility.^21^

Managing low testosterone in men who wish to remain fertile is problematic and a randomized control trial (RCT) of leflutrozole in obesity associated hypogonadotrophic hypogonadism demonstrated normalization of total testosterone (TT) in obese men. Follicle-stimulating hormone (FSH) & Luteinizing hormone (LH) and semen parameter changes support that leflutrozole may preserve/improve testicular function.^22^

Ejaculatory dysfunction encompasses several disorders related to DM and its complications, such as premature ejaculation (PE), anejaculation (AE), delayed ejaculation, RE, ejaculatory pain, anesthetic ejaculation, decreased ejaculate volume, and decreased force of ejaculation.^23^ The problems linked to ejaculatory dysfunction may lead to inferior quality of life as both AE and RE are alleged to alter their fertility potential. The initial management for PE is controlling the patient’s blood glucose that in some cases may allow recovery the normal ejaculatory function. Amelioration of glycemic variability would improve PE in type 1 diabetes patients. In cases of concomitant erectile disorder (ED) and PE, ED should receive phosphodiesterase type 5 inhibitors before, or at least at the same time as PE. The efficacy of the combined use of phosphodiesterase type 5 inhibitors and dapoxetine in males with comorbid PE and ED are supported by some studies. Several drugs are recognized for the possible use in DE/AE. Those agents include testosterone, amantadine, cyproheptadine, cabergoline, bupropion, yohimbine, buspirone, bethanechol, and others. Yet, no drug has been approved for this indication. Infertility is usually the main concern in RE patients as the combination of dry orgasm and infertility makes the condition upsetting to the patient and his partner. Therefore, many lines of therapeutic approaches are advocated, either medical or surgical, with limited success rates.

The processes of ejaculation and orgasm are complex and include neuronal and hormonal factors exacerbated by underlying diabetes, as well as psychological and interpersonal dynamics. Care of the patient presenting with a potential Disorders of Ejaculation and Orgasm centers on sensitive history taking and selective testing. Declines in semen volume may occur naturally with age and can be seen in the context of medical or surgical therapies. Pain with ejaculation/orgasm has a myriad of potential etiologies and may be part of a complex chronic pelvic pain syndrome; assessment for related diagnoses that may be contributory is warranted.^24^

SD is commonly encountered in older T2DM patients and diabetic kidney disease (DKD) affects almost half of them. The eGFR has been significantly associated with SD, ED, and FSD, while SD and ED were proven to be significant determinants for the eGFR levels.^25^ Diabetic nephropathy (DN) is the leading cause of end-stage renal disease. Preclinical and experimental studies show that PDE5 inhibitors (PDE5i’s) exert protective effects in DN improving perivascular inflammation.^26^^,^^27^

Female sexual dysfunction (FSD) is more common among women with type 1 diabetes than among women without type 1 diabetes. Patients with type 1 diabetes should be questioned in terms of sexual health. Health professionals should give more attention to and provide guidance regarding sexual function in both men and women with type 1 diabetes.^28–36^

#### Predictors of SD in CVD, diabetes, and obesity

Growing evidence suggests that the a-Klotho gene has a crucial role in the control of multiple metabolic processes and the pathophysiology of common aging-related disorders. Higher glycoprotein S-Klotho plasma levels are associated with a lower risk of CVDs, type 2 diabetes incidence, and all-cause mortality. Free testosterone, lean body mass, and physical fitness are linked to S-Klotho plasma levels in middle-aged adults.

S-Klotho plasma levels have been associated with sexual desire and sexual function in men and, also, with dyadic sexual desire and sexual function in women. S-Klotho plasma levels may represent a potential new biomarker for sexual desire and sexual function. The link to lean body mass may be related to benefits in sexual desire and sexual function.^37^

Growing evidence suggests that the FGF-Klotho endocrine system also has a crucial role in the pathophysiology of ageing-related disorders, including diabetes, cancer, arteriosclerosis, and chronic kidney disease. Therefore, targeting the FGF-Klotho endocrine axes might have therapeutic benefit in multiple systems; investigation of the crystal structures of FGF-Klotho-FGFR complexes is paving the way for the development of drugs that can regulate these axes and treat SDs.^38–46^

#### Metabolic actions of testosterone in relation to diabetes

Testosterone enhances insulin sensitivity in obese men with hypogonadism by decreasing fat mass, increasing lean mass, decreasing free fatty acids, and suppressing inflammation. At a cellular level, testosterone increases the expression of insulin receptor β subunit, insulin receptor substrate-1, protein kinase B and glucose transporter type 4 in adipose tissue and adenosine 5′-monophosphate-activated protein kinase expression and activity in skeletal muscle. Observational studies show that long-term therapy with testosterone prevents progression from prediabetes to diabetes and improves HbA1c.

Testosterone increases skeletal muscle satellite cell activator, fibroblast growth factor-2 and decreases expression of the muscle growth suppressors, myostatin, and myogenic regulatory factor. Testosterone increases hematocrit by suppressing hepcidin and increasing expression of ferroportin along with that of transferrin receptor and plasma transferrin concentrations. Testosterone also increases serum osteocalcin concentrations, which may account for its anabolic actions on bone. In conclusion, testosterone exerts a series of potent metabolic effects, which include insulin sensitization, maintenance, and growth of the skeletal muscle, suppression of adipose tissue growth and maintenance of erythropoiesis and haematocrit.^47^

Two years of testosterone therapy (TTh) resulted in normalized serum testosterone levels, improved glycemia, endothelial function, lipids, and insulin sensitivity, and improved the symptoms of hypogonadism, potentially reducing cardiovascular risk in obese men with FH and T2D. This is supported by multiple studies.^48–51^

What emerges from the current clinical literature is that, irrespective of the length of study durations, TTh provides significant health benefits and reduces the risk of CVD. More important is that data from many observational and registry studies demonstrated that longer durations of TTh were associated with greater health benefits and reduced cardiovascular risk. T therapy in men with T deficiency reduces the incidence of major adverse cardiovascular events attributed to improving overall metabolic function.^52^

In men with hypogonadism and preexisting or an elevated risk of CVD, testosterone-replacement therapy was noninferior to placebo with respect to the incidence of major adverse cardiac events.^53^^,^^54^

#### Obesity

Obesity is a growing public health concern worldwide, and results in increased risk of CVD, type 2 diabetes, metabolic syndrome, insulin resistance, dyslipidemia, hypertension, sexual problems, and reduced sex hormone production.^55–61^

##### Impact of weight loss

Obesity is associated with hypogonadism, SD, and impaired fertility in men. Bariatric surgery improves male and female sexual health. It is associated with significant increase in International Index of Erectile Function (IIEF) score and serum testosterone levels. Similar benefits in sexual function and self-esteem are seen in women.^62–68^

Several papers confirm that exercise induces a series of cardiovascular, metabolic, and psychological adaptations that protect against erectile dysfunction (ED) in men and SD in women and in addition improves sexual desire and sexual function in women.^28^^,^^69–71^

Both low and high physical activity levels were associated with more than 20% reduction in the risk of ED in men aged 40 years or older.^72^

In addition, research supports the utility of encouraging healthy lifestyle habits among young men to reduce the subsequent risk of prostatic enlargement and ED.^73^

A study illustrated the potential value of promoting Sexual Activity (SA) support for both patients who are post-acute coronary syndrome and their partners and reports that SA support provided at clinical review would be viewed as important, needed, and acceptable.^74^

The potential of the oxytocin system as a behavioral and molecular target for the prevention and treatment of CVD is of interest as it is associated with sexual activity. Focus is put on the affiliative and sexual significance and the different options and limitations associated with a pharmaceutical approach.^75^

Postmenopausal women, particularly those in the middle-age range, should be assessed for CV risk factors and FSD, SD is a harbinger of CVD in postmenopausal women. Identification is important so that both CVDs and sexual problems do not persist unnoticed.^76^

ED is a hallmark of CVD and includes a family history of cardiometabolic events, alcohol abuse, fatherhood, decreased partner’s sexual interest, severe impairment in erection during intercourse or during masturbation, impaired fasting glucose, increased triglycerides, obesity even without metabolic complications, decreased penile blood flows, or impaired response to an intracavernosal injection test. Recognizing these risk factors may help in identifying, among subjects with ED, those who merit stricter lifestyle or pharmacological interventions to minimize their CV risk. Effective correction of risk factors in ED men considered as elevated risk, besides reducing CV risk, is also able to improve erectile function.^77^

Men with priapism are at increased risk for cardiovascular and cerebrovascular events in the years following a priapism.^78^

#### The impact of the new anti-diabetes medications on sexual function

Several aspects of altered metabolism in individuals with T2D may help to compromise the penile vasculature structure and functions, thus exacerbating the imbalance between smooth muscle contractility and relaxation. Among these, advanced glycation end-products and reactive oxygen species derived from a hyperglycemic state are known to accelerate endothelial dysfunction by lowering nitric oxide bioavailability, the essential stimulus of relaxation. The complications of DM represent a serious health problem. The long-term complications include macroangiopathy, microangiopathy, and neuropathy as well as SD in both men and women. ED has been considered the most important SD in men with DM. The prevalence of ED is approximately 3.5-fold higher in men with DM than in those without DM. Common risk factors for the development of DM and its complications include sedentary lifestyle, overweight/obesity, and increased caloric consumption. Although several studies have explained the pathogenetic mechanisms involved in the generation of erectile failure, few studies to date have described the efficacy of glucose-lowering medications in the restoration of normal sexual activity. The role of different anti-diabetes therapies in the potential remission of ED needs to be explored, highlighting specific pathways whose activation or inhibition could be fundamental for sexual care in a diabetes setting. Randomized clinical trials are needed to provide evidence supporting the use of Glucagon-Like Peptide-1 Receptor Agonists for treating ED in T2D.^79^

The Mediterranean diet was effective in most studies for the protection of erectile function. Furthermore, anti-hyperglycemic drugs seem to show an overall protective role on erectile function.^80^

SGLT2i should be considered for a trial course of therapy in patients with TDM with ED. The select group of patients who are contraindicated for PDE5i would also gain from this novel approach. The improvement in the microvascular circulation with enhanced vascularity could be the mechanistic reason for the observed clinical benefits.^81^^,^^82^ A better glyco-metabolic profile, as well as new antihyperglycemic drugs, seem to have a positive effect on ED.^83^

Liraglutide exerts protective effects on ED associated with the regulation of smooth muscle dysfunction, oxidative stress, and autophagy, independently of a glucose- lowering effect. It provides new insight into the extra pancreatic actions of liraglutide and preclinical evidence for potential treatment for DMED.^84^

Liraglutide produced a significant increase in serum levels of testosterone (TT), free testosterone (Tl), and possibly sex hormone-binding globulin (A); in addition to improving the sexual function and quality of life of women. Liraglutide could become a promising drug for the treatment of obesity, achieving positive effects on sexual function and quality of life in women.^85^

Giagulli et al.^86^ evaluated the effect of liraglutide in obese, hypogonadal patients with T2DM who received metformin and testosterone replacement. The group with liraglutide showed greater benefits on erectile function as well as increased testosterone levels.

Evidence from animal models suggests that DPP4i drugs could improve erectile function in patients with T2DM, promoting vascular repair and endothelial function^87^ and improving the effects of vasorelaxants mediated by vascular endothelial growth factor.^88^

#### Lifestyle modifications and glycemic control

In a systematic review and meta-analysis including 740 participants from four countries, lifestyle modifications aimed at decreasing cardiovascular risk demonstrated an improvement in sexual function.^89^ Several RCTs analyzing the effects of reduced caloric intake, better food quality with a Mediterranean diet, and increased physical activity reported improvements in metabolic parameters, anthropometric measurements, and sexual function.^90^^,^^91^

A weight loss of 5% from the initial weight has been the minimum recommended weight loss percentage for clinical benefit. Greater weight loss leads to even greater benefits in terms of reducing BP; improving low-density lipoprotein and high-density lipoprotein levels and insulin resistance and reducing the number of medications to control DM, hypertension, and dyslipidemia^92^^,^^93^ As previously reported, weight loss is strongly recommended to improve erectile function considering that it can increase testosterone levels mainly by improving testicular function and reducing conversion of testosterone to β-estradiol via aromatase activity in the adipose tissue, as well as increasing Sex Hormone Binding Globulin (SHBG) concentration as a consequence of reduced insulin levels.

Physical exercise is strongly recommended for both patients with T1DM and T2DM given its contribution to weight loss, improved blood glucose control, reduced cardiovascular risk, and improved well-being and sexual function. While physical exercise may improve serum testosterone levels, high-intensity exercise can also be counterproductive.^92–99^

Despite being used for ED, PDE5’s clearly have favorable effects on the cardiovascular system, primarily due to the restoration of the nitric oxide (NO) cyclic guanosine monophosphate (cGMP) protein kinase G (PKG) (NO-cGMP-PKG) signaling pathway and amelioration of the pro-inflammatory state that characterizes T2DM and the consequent endothelial dysfunction and increased cardiovascular risk. Although results from preclinical and human studies have been promising, additional studies are needed to determine the role of PDE5is in cardiovascular protection in T2DM.^100^

### Section B: Chronic pain

#### Introduction

CP is recognized as a prevalent global health concern often associated with significant disability and a noteworthy psychological and social impact.^101^ According to the International Association for the Study of Pain (IASP), CP is characterized by persistent sensory and emotional discomfort linked to actual or potential tissue damage.^102^ Prevalence rates of CP vary from 11% to 40%, with a study by the US Centers for Disease Control and Prevention estimating it at 20.4%.^103^ CP conditions encompass physical injuries, arthritis, neuropathies, musculoskeletal issues, and other etiologies.

Recent research on CP and sexuality has particularly focused on conditions such as chronic migraine (CM), fibromyalgia (FM), and irritable bowel syndrome (IBS), which are more prevalent in women than men. Additionally, in the last years, there has been an increase in literature regarding sexuality in arthritis rheumatoid (AR), genitopelvic pain-related conditions (eg, vulvodynia, vaginismus, endometriosis), and multiple sclerosis. Critical discussions in this context will primarily draw from studies on CM, FM, IBS, and AR, but not limited to these conditions. Gynecological conditions and multiple sclerosis are out of the scope of the current review and will be addressed separately in specific guidelines.

#### Innovation in CP treatment and sexual function

##### SDs and associated CP symptoms

About rates of SD, a lot of recent literature shows data that is often not comparable in terms of methodology and measurement used. Most of the studies assess sexual functioning through self-report questionnaires such as FSFI and IIEF.^104^^,^^105^ This gives us a limited view without considering the classification criteria of SD. Studies showing a prevalence of SD in CP range from 35% to 97%,^106–122^ with women appearing to be more affected than men. The most affected areas seem to be desire and sexual pain for women and arousal for both women and men.

This high presence of sexual impairment may be due to a multifactorial cause involving the CP condition per se, associated symptoms, treatments used, and relational and social experience of the condition.

People with CP often experience a range of physical, emotional, and psychological symptoms such as anxiety, depression, fatigue, difficulty sleeping, irritability, difficulty concentrating, and loss of interest in daily activities. Several studies have found a negative association between sexual functioning and depression, low sleep quality, anxiety, fatigue, and pain-related interference in life (Level 2 of evidence).^123^ The magnitude and strength of each of these associations were quite similar across gender and CP type. In addition, depression was identified as the most consistent negative predictor of sexual function across studies, followed by anxiety (Level 3 of evidence).^109^^,^^124^

##### Controversies in opioid therapy

Despite the demonstrated efficacy of opioid therapy in alleviating symptoms of CP, numerous studies report a range of significant side effects, although the evidence is not unanimous. Patients undergoing opioid treatment exhibit a heightened likelihood of experiencing constipation, dizziness, drowsiness, fatigue, hot flashes, increased sweating, nausea, itching, vomiting, and urinary retention compared to those receiving a placebo in clinical trials (Level 1-3 of evidence).^125–127^ While not related to sexuality, these factors can exacerbate sexual experiences in individuals with CP.

Men commonly experience opioid-induced hypogonadism, reduced sperm production, and alterations in testicular interstitial fluid as notable sexual and reproductive consequences (Levels of 1 and 2 of evidence).^128^^,^^129^ Similarly, women may encounter heightened vaginal dryness, infertility, irregular menstruation, and intensified menopausal symptoms, both in short and long-term opioid usage scenarios (Level 2 of evidence).^130^ Overall, opioid users are more prone to sexual dissatisfaction and issues, particularly pertaining to diminished sexual desire, erectile difficulties, premature/delayed ejaculation, painful intercourse, and difficulty achieving orgasm (Level 2-3 of evidence).^131^^,^^132^ This trend is corroborated by the increased utilization of medications to address SD among opioid users (Level 1 of evidence).^133^

##### Controversies in antidepressant therapy

Antidepressants are frequently used in the management of CP, particularly in FM, diabetic neuropathy, or chronic back pain. Antidepressants can effectively enhance CP management by reducing pain perception, possessing anti-inflammatory properties, and influencing sleep and mood. However, selective serotonin reuptake inhibitors (SSRIs) and serotonin-norepinephrine reuptake inhibitors like duloxetine, desipramine, or fluoxetine have been associated with challenging outcomes concerning patients’ sexual health. Specifically, women with FM who use antidepressants have reported decreased levels of sexual desire, both in partnered and solitary contexts, compared to non-users (Level 4 of evidence).^134^ Additionally, SSRIs are commonly linked with various adverse side effects that may indirectly affect sexual experiences, including headache, dry mouth, nausea, fatigue, dizziness, sweating, nervousness, and difficulties with urination (Level 1 of evidence).^135^^,^^136^ Nonetheless, due to the limited amount of scientific evidence available, it remains challenging to determine the extent to which antidepressant therapies directly impact sexuality versus inherent features of CP.

##### HCPs and CP: advances in integrated approaches and residuality of sexual health

The literature presents a uniform scenario characterized, on one hand, by CP patients who refrain from discussing their sexuality with HCPs, despite acknowledging that it could enhance guidance and support in managing their sexual issues (Level 3 of evidence).^137^ Additionally, patients lament the lack of visibility and information regarding sexuality and treatment side effects (Level 3 of evidence).^110^ On the other hand, HCPs often avoid exploring patients’ sexual and relational concerns due to taboos, lacking specific training, or overlooking the significance of sexual well-being and pleasure (Level 3 of evidence).^138^^,^^139^ This situation creates an area of “unmet need” (Level 1-4 of evidence).^115^^,^^140–143^

It is increasingly imperative for HCPs to recognize the significance of erotic expression and the potential for pleasure among CP patients despite their pain (Level 4 of evidence).^144^ Understanding the interplay among these factors is vital for a more comprehensive assessment and management of CP (Level 4 of evidence).^145^ Patient care should adopt a multidisciplinary approach to address the various levels of complexity in patients’ health, encompassing their sexual expression (Level 4 of evidence).^144^^,^^146^

Similarly, providing HCPs with tools to sensitively address this aspect and acknowledge their sexual and reproductive rights is crucial (Level 4 of evidence).^144^ In addition to standard clinical measurement tools, it is advisable to incorporate questionnaires assessing sexual health (Level 2 of evidence).^147^ Moreover, initial evidence suggests that utilizing the ReConnect model in both individual and group sessions may aid HCPs in supporting their patients’ sexual activity goals (Level 3 of evidence).^148^

##### Relationship and sexual expression issues

Most contributions come from qualitative studies (interviews and focus groups), more able to capture specific nuances of the sexual and relational experience of people with CP. Although there is considerable variability in how CP affects sexual and relational well-being, a key point is the significant prevalence of dissatisfaction with sexual life, particularly among older patients (Level 2 of evidence).^132^^,^^149^

CP affects sexual identity, body image, and self-worth (Levels 2-4 of evidence).^146^^,^^150^^,^^151^ On the relational side, Ragab et al.^152^ (Level 3 of evidence) sexual relationships are impacted in 86% of migraine patients, with a rate of 26% of divorce. In this sense, problems are not limited to sexual life or to the patient but also involve their partners (Level 3 of evidence).^153^ Interference in sexual life may create tension, anxiety, and emotional stress inside the relationship (Level 4 of evidence)^144^ since sexuality is often seen as the “glue” holding the relationship together (Level 4 of evidence).^142^

The study by Ferrari et al.^141^ (Level 4 of evidence) highlights the four main domains affecting the sexual and relational experience of CP patients: difficulty in allowing sexual activity; suffering from loss of pleasure and sexual identity; need for mental and physical support from a partner to maintain a sexual activity; need for support from HCPs to preserve their sexual life. In this sense, for certain couples, the illness serves as an opportunity to reinforce their bond, prompting the partner to adopt a supportive, non-judgmental, and proactive approach. Effective communication and the exploration of alternative forms of intimacy may facilitate this empowerment, enabling couples to adapt and discover new strategies to sustain their sexual lives despite experiencing pain (Level 4 of evidence).^143^

### Section C: Breast cancer

#### Introduction

BC remains one of the most common malignancies in women, and the interventions to possible contain or cure disease (radiation, chemotherapy, cytostatic medications with and without chemically induced menopause) have far reaching implications on the female sexual response cycle. With advances in diagnostic techniques and genetic assessment of those at risk, there is a lead time bias which contributes to earlier diagnosis of younger women, and more survival time in the post therapeutic time. The intersection between BC and sexual function is a dynamic field of study and has been the focus of much research in the past decade. Due to limited content capacity, the primary focus for this intensive review will highlight innovations and updates on Genito-urinary Syndrome of Menopause (GSM), position statements, and controversies. New medications and novel device interventions will also be featured. The importance of sexual orientation, gender identity and ethnic diversity when discussing BC sexuality cannot be underscored yet is beyond scope of this concise review.

#### Moisturizers and lubricants

Vaginal moisturizers (maintain hydration and use is independent of intercourse) and lubricants (used during coitus to increase lubricity) are considered the frontline mainstay treatment for vaginal dryness and painful intercourse in women with BC and much focus over the last few years has been on the concepts of vaginal pH, osmolality, and the vaginal biome. Palacios et al.^154^ demonstrated that with reformulated ingredients to maintain the World Health Organization new lubricant standards with vaginal pH and osmolality (≤1200 mOsm/kg)^155^ water-based lubricants are effective and well tolerated to relief vaginal dryness. While there remains, limited information comparing water based versus anhydrous, silicone-based lubricant, Hickey et al.^156^ randomized double blind cross over trial in breast cancer patients (BCPs) demonstrated that water- and silicone-based lubricants did not differ statistically in efficacy based on total sexual discomfort, but silicone was preferred over water based, and total discomfort was lower with silicone use. Polycarbophil-based vaginal moisturizers, can create a film over the vaginal tissues which remain adherent to the epithelial cells thereby improving objective symptoms of dryness, dyspareunia, and sexual function/satisfaction,^157^^,^^158^ without impact endometrial thickness in BCP.^159^ Juliato et al.^160^ confirmed that polyacrylic acid can help improve sexual function in BCP on tamoxifen. Advani et al demonstrated that a moisturizer/lubricant coupled with counseling and dilator helped women maintain stable sexual function on aromatase inhibitors.^161^ Sexual accessories (toys) or vaginal dilators, which are made of silicone, should not be used with silicone-based moisturizers or lubricants.

Many new novel non-hormonal products are under varying stages of study and investigation. A novel oil-in-water emulsion vaginal cream demonstrated efficacy and safety for BCP undergoing treatment in a limited 4-week, non-randomized trial.^162^ There remains scant information on non-hormonal vaginal products and their specific implications on the vaginal biome of BCP.

#### Professional organizations guidelines/consensus or reviews

Many professional organizations, such as joint consensus report from The Menopause Society (NAMS) and the International Society for the Study of Women’s Sexual Health (ISSWSH)^163^; The American College of Obstetricians and Gynecologists,^164^ 2021; American Cancer Society/American Society of Clinical Oncology Breast Cancer Survivorship Care guideline ^165^^,^^166^; and the International Society for the Study of Vulvovaginal Disease^167^; have published statements concerning the management of GSM associated with BC. In addition, NAMS has their own 2020 position statement.^168^ See key recommendations. The NAMS/ISSWSH, ACOG and other guidelines, and detailed literature review articles^169–178^ discuss and advocate nonpharmacological interventions such as: education, counseling/sex therapy, moisturizers/lubricants, self-stimulators/vibrators, dilators, and physical therapy as key components for front line interventions. Many review articles^169–178^ also suggest minimally absorbed local vaginal estradiol for refractory cases where symptoms are unrelieved. Ospemifene and Intravaginal dehydroepiandrosterone (DHEA) may also be used but long-term safety data are lacking. In a recent large claims-based analysis, Agarwal et al.^179^ did not find an increased risk of BC recurrence in women with a personal history of BC who were using vaginal estrogen for GSM. Vaginal Prasterone (DHEA) was studied in a small open prospective pilot study (VIBRA pilot) and^180^ demonstrated that serum estradiol levels remained low after 6 months of follow up while sexuality and vaginal health improved significantly. The authors concluded that Prasterone seems a safe and effective treatment option for BCP on AI who have GSM. Recent emerging controversies regarding the use of minimally absorbed local vaginal estrogen,^181^ neither vaginal nor systemic vaginal hormone therapy was associated with an increase of recurrence or mortality. A subgroup analysis, however demonstrated an increased risk of recurrence but not mortality in patients on vaginal estradiol therapy with adjunctive AI use. While intravaginal DHEA and minimally absorbed local vaginal estradiol remain unapproved in this patient population, it is critical to note that most sexual medicine specialists unanimously believe that low dose vaginal estrogen, and Prasterone, appear perfectly appropriate, beneficial, and safe in this setting. Further, and despite some outlier papers, as mentioned above, suggesting that such treatment may not be safe, most of the best literature is extremely reassuring on this point. The decision to use these medications should be based upon shared decision making between the patient, her oncological team, and her sexual health specialist.

Despite extensive reviews and consensus guidelines on the treatment of GSM on BCP, HCP (OB GYN/Primary care/Oncologist have low comfort for managing GSM and prescribing BCP vaginal estrogen.

#### Device treatment and BCP

There has been a plethora of published systematic reviews and meta-analyses concerning the efficacy and safety of CO2 laser for the treatment of GSM in BCPs.^182–191^

Cucinella et al.^182^ provide a current review and is an excellent summary that illustrates that most data is derived from single arm studies which are prospective; the data from randomized sham-controlled trials are limited ^192^^,^^193^ and while efficacy and safety for both fractional and Erbium laser ^194^^,^^195^ the short term are promising, further studies with longer durations of follow-up are necessary.

The scarce scientific research on radiofrequency use in the BCP for the treatment of GSM, has demonstrated extremely limited efficacy if any and mostly short-term safety surveillance.^196–198^

It remains important to mention that most novel devices used in BCP have shown efficacy (in improving vaginal dryness, dyspareunia, and sexual function measures). Tolerability and safety profile remains positive with limited side effects commonly reported as transient burning, procedural pain, and/or probe insertion discomfort.^199^ However, these procedures remain cash based, uncovered by medical insurance within the USA but may vary elsewhere and unapproved by regulatory bodies. The data are inconsistent, and many clinical trials remain nonrandomized, without shams, and are observational, retrospective, and are of small numbers, with varying design/protocols and have varying degrees of follow up. Further in-depth study and rigorous research is suggested and warranted. Consumer and health care professionals should exercise vigilance and remain wary of cash-based medi-spas that strongly advocate for these interventions over other simpler approved medications and or therapeutic interventions.

#### Testosterone

Although it is not feasible to perform an extensive review of testosterone use (pellets/injections/intravaginal cream) in this short summary, it is worthy to note that there is emerging data to support subcutaneous testosterone use for reduction in incidence of BC^200^^,^^201^ and that testosterone may improve symptoms of lowered libido and dyspareunia.^201–204^ Caution should be exercised as there is limited safety and long-term use data.

#### Internet-based interventions

There is growing interest in internet-based survivorship care planning (SU) cognitive behavioral therapy (CBT) for interventions that improve sexual function in BCP. Hummel et al demonstrated that internet-based CBT has beneficial effects on sexual function, body image, and menopausal symptomatology in BCP.^205^ Gorman et al. demonstrated that virtual mindfulness-based intervention is acceptable for BCP.^206^

### Section D: Gynecological cancer

#### Introduction

Gynecologic cancers, which include endometrial, cervical, ovarian, vaginal, and vulvar cancers, are among the most common cancer in women.^207^ Collectively, data from recent systematic reviews suggest that gynecologic cancers can lead to significant sexual sequelae,^208–211^ with gynecologic cancer patients reporting worse sexual function than women in the general population 1-5 years after cancer diagnosis.^212^ Sexual problems related to treatment for gynecologic cancers are common, multi-faceted,^208^ and considerable, with two-thirds to three-quarters of women treated for gynecologic cancer reporting decreased sexual activity^213^^,^^214^ and over half reporting decreased sexual satisfaction.^213^

#### Overview of sexual problems for gynecologic cancer survivors

The nature and severity of sexual sequelae in gynecologic cancer survivors depend on the cancer site and type and extent of treatment offered, including the extent of surgery and whether the surgery is nerve-sparing,^212^^,^^215^^,^^216^ if systemic therapy or pelvic radiation therapy are given,^214^ which combination of treatments are given,^217^ as well as other factors, such as partnered status^218^ and disease stage^212^; women with advanced disease have worse sexual function than those diagnosed with gynecologic cancer at an earlier stage.^212^^,^^217^ Ovarian insufficiency, which can result from pelvic surgery, radiation, or chemotherapy, can lead to significant sexual problems related to early or abrupt menopausal symptoms.^219^ A recent meta-analysis of studies examining SD in patients with cervical cancer reported an overall incidence of SD of 80%,^210^ and similar rates have been reported for women with ovarian cancer.^220^ When using the more stringent DSM-5 diagnostic criteria for FSD,^221^ a recent study found a prevalence of 44% in women with gynecologic cancer.^213^ Risk-reducing surgery for ovarian cancer and, to a lesser extent, hysterectomy, can significantly affect women’s sexual function, whereas use of hormone replacement therapy can mitigate some of these effects and make sex more comfortable.^222^^,^^223^

#### Sexual problems in diverse subgroups of gynecologic cancer survivors

Although much of the literature examining sexual function in patients with gynecologic cancer has historically suffered from the limitation of largely white and well-educated patient samples,^224^ a recent trend in research in this area suggests efforts are underway to change this. For instance, in the past few years, several review articles have focused on the unique concerns of female cancer survivors who identify as women of color^225^ or in older cancer populations, including those with gynecologic cancer.^226^ Findings from these studies have suggested increased effects of treatment on body image and femininity in Latina women, the unique role of spirituality in coping in African American women,^225^ and the need to address taboo associated with discussing sexual issues in Muslim women with gynecologic cancer.^227^ A 2015 study examining sexual problems in 243 medically underserved patients with gynecologic cancer found that lack of a partner was among the most commonly cited reasons for lack of sexual activity, which was high in this sample and comparable to those reported more broadly.^224^ Taken together, these studies find similar sexual complaints in patients from various races/ethnicities, ages, and religious backgrounds, while also pinpointing concerns unique to these populations.

#### Pelvic floor dysfunctions

Pelvic floor dysfunctions (also referred to as pelvic floor disorders [PFDs]) include symptoms related to urinary and bladder symptoms (eg, incontinence, urinary tract infections), SDs (eg, dyspareunia, obstructed intercourse), bowel symptoms (eg, incontinence, constipation), pelvic organ prolapse, and pelvic pain.^228^ Recent systematic reviews of PFD in patients with gynecologic cancers found that these symptoms are highly prevalent,^229^^,^^230^ with SDs, vaginal dryness, stenosis, and pain among the most common sexual side effects.^230^^,^^231^ In cervical cancer, recent data provide evidence for distinct changes in the vagina and vaginal epithelium after radiation therapy, including reduced vaginal epithelial volume and atrophy^232^; changes such as these, in turn, are related to reduced vaginal lubrication, loss of vaginal elasticity, and reduced vaginal length and swelling in the genitals occurring during sexual arousal.^232^

#### Clinical communication about sexual health in care of gynecologic cancer patients

Although the majority of women with gynecologic cancer believe that sexual function is important to their overall health and believe their providers should ask about these issues,^233^ most report never having had a discussion with their providers about sexual issues.^234^ Only around 20% of gynecologic cancer providers have received training in discussing sexual function,^235^ suggesting inadequate clinician knowledge and training in how to discuss these issues appropriately and effectively is a major barrier to communication about this issue. Additional communication barriers include clinician discomfort, lack of time, and certain beliefs, including that patients will raise the topic if needed.^235^^,^^236^ Discussions of sexual issues with gynecologic cancer patients should include open, honest communication, reputable information, and resources provided to patients, and the timing of information with patients’ preferences.^237^

While efforts to address cancer clinicians’ knowledge and training in discussing sexual health are critical, patient-focused approaches to prompt patients to raise sexual concerns according to their needs and preferences are growing.^238–243^ One recent example is a patient-focused multimedia intervention that was recently found feasible and acceptable and showed promising effects improving gynecologic cancer patients’ self-efficacy and communication with respect to sexual health.^244^ If shown to be effective in a larger trial that is currently underway, this intervention could be disseminated widely to facilitate integration of sexual health into cancer care in this population.

#### Interventions addressing sexual problems in gynecologic cancer

##### Overview

A recent systematic review of interventions to manage SDs in women with cancer included 36 studies, of which only four were conducted in gynecologic cancer samples; three of these were in mixed breast or gynecologic cancer samples, and one was conducted in an ovarian cancer sample.^245^ Unfortunately, of the 14 randomized controlled trials of interventions included in the review, only three included gynecologic cancer patients whereas most were in BC, suggesting a significant need for randomized controlled trials of interventions in gynecologic cancer relative to BC.

Pelvic Floor Rehabilitation Interventions. A systematic review and meta-analysis from 2020 on evidence for non-medical muscle interventions on PFDs in patients with gynecologic cancer found five RCTs and 2 retrospective cohort studies examining these interventions.^246^ Results showed support for pelvic floor muscle training alongside counseling and yoga or core exercises in improving patients’ sexual function in cervical cancer survivors,^246^ and studies conducted since that review was published in 2020 find further evidence supporting the use of pelvic floor physical therapy on sexual function.^247^ Regarding vaginal dilators alone, a randomized controlled trial conducted in 88 gynecologic cancer patients undergoing gynecologic brachytherapy found no significant differences in vaginal length, width, or area in patients who used vaginal dilators for 3 months as compared to a care-as-usual control group^248^; when stratified by adhesion, however, the control group saw a significant decrease in the vaginal area, and greater reduction in vaginal dryness and other pelvic floor symptoms in the dilator group only.^248^ Adding a psychoeducational booklet increased vaginal dilator use in women receiving pelvic radiation therapy for gynecologic or anorectal cancer, but did not have effects on women’s sexual function.^249^ Encouragingly, recent data find positive rates of patient engagement with pelvic floor therapy interventions,^250^ and the ability to train nurses to deliver sexual rehabilitation interventions including vaginal dilation,^251^ which may help increase the uptake of these interventions. Taken together, findings indicate that pelvic floor rehabilitation and vaginal dilators alone are recommended (Grades A and B, respectively).

##### Topical interventions

Relative to BC, there have been comparatively few high-quality trials evaluating topical interventions in the treatment of vaginal symptoms affecting sexual function in patients with gynecologic cancer. One of the largest such trials was a three-arm RCT led by Barton^252^ that compared two dosages of vaginal DHEA against plain vaginal moisturizer for alleviating vaginal symptoms in 464 women with either a breast or gynecologic cancer history reporting at least moderate vaginal symptoms. Findings revealed that all arms reported improvement in severity of dryness and dyspareunia, but the higher dose of DHEA led to greater improvements in sexual function as compared to the lower dose and moisturizer. Only 3% of the sample had gynecologic cancer, however, making it difficult to draw conclusions on the efficacy of this intervention, specifically in this population. Further rigorous studies with larger gynecologic cancer samples are needed to bolster recommendation strength (Grade D recommendation).

##### Pharmacological interventions

As with the topical agents, the research for pharmacological treatments in women with gynecologic cancer is also scarcer relative to BC. A 2022 trial by Barton^253^ evaluated two dosages of bupropion, a dopaminergic agent, in a randomized, placebo-controlled trial in 230 women with a history of non-metastatic breast or gynecologic cancer reporting low sexual desire and found that bupropion was no more effective than placebo in improving sexual desire (Grade D recommendation). Ospemifene, an estrogen receptor agonist/antagonist, also known as a selective estrogen receptor modulator selective estrogen receptor modulator, had previously demonstrated benefit in improving vulvo-vaginal atrophy in postmenopausal women without a cancer history,^254^^,^^255^ and was more recently assessed in a sample of 52 women with a diagnosis of early-stage cervical cancer.^256^ Results of the single arm trial suggested that vaginal health and sexual function outcomes improves significantly.^256^ However, to date, no large randomized controlled trials of ospemifene in gynecologic cancer patients have been conducted, leading to limited ability to draw conclusions about the efficacy of this medication in this population (Grade D recommendation).

##### Fractional CO2 lasers

Microablative fractional CO2 lasers, which are believed to promote tissue regeneration within the vaginal wall, have been evaluated as an additional approach to managing vulvo-vaginal atrophy in patients with cancer. A systematic review of fractional CO2 lasers conducted in 2022 surveyed nine studies of this treatment for female cancer survivors, the vast majority of which were conducted in BCP samples.^257^ However, only one study included gynecologic cancer patients and that was a retrospective study which included both patients with breast and gynecologic cancers (18% gynecologic cancer). Although significant improvements were seen over time in vaginal dryness, dyspareunia, and other vaginal symptoms, because most of the sample had BC, there is limited data supporting this treatment for gynecologic cancer patients (Grade D evidence).^258^

##### Psychoeducation and behavioral interventions

A 2022 systematic review of RCTs of six psychoeducation interventions in gynecologic cancer patients and survivors found that these interventions overall improved sexual function in patients, with more substantial effects seen when the partner was involved.^259^ However, effects varied, with some of the studies finding less substantial effects on patients’ sexual outcomes.^260^ Different approaches have also shown promise in uncontrolled studies, including a 12-week psychoeducational program featuring mindfulness meditation training for gynecologic and colorectal cancer survivors, which found significant increases over time in sexual function and distress,^261^ a single half-day mixed educational and behavioral sexual rehabilitation intervention for ovarian cancer survivors that found significant effects on patient sexual outcomes to 6-month follow-up,^262^ and an online self-help intervention for patients with breast or gynecologic cancer, which saw improvement in sexual outcomes but low uptake.^263^ There is thus mounting evidence for benefits of psychoeducational and behavioral interventions in this population, and because these interventions generally do not incur side effects or other negative risks, they are highly recommended (Grade A recommendation). Exercise interventions were reviewed for gynecologic cancer survivors in 2016, and at that time, there was insufficient data available to assess effects on sexual function.^264^ In a more recent review, one single-arm physical activity trial demonstrated improvements in sexual function for endometrial cancer survivors at 6-month follow-up^265^ (Grade D recommendation).

### Section E: Oncologic interventions

#### Introduction

In 2040, the expected global number of cancer cases is expected to reach 28 million.^266^ Oncologic outcomes continue to improve, with an increasing number of cancer patients surviving 5 years or more. Thus, it remains imperative to improve the therapeutic index of curative therapies about health-related quality of life (QOL) by reducing treatment-related toxicities. Sexual functioning is an essential component of human health across the lifespan, therefore maintaining sexual function after cancer treatments is an important priority. Herein, we review advancements integrating primary prevention of SD due to cancer treatment, by describing innovations and updates in oncologic interventions that preserve sexual function in cancer survivorship.

#### Innovations in oncologic interventions and sexual function

##### Surgical interventions

###### Bladder

Cystectomy is a preferred treatment for muscle-invasive bladder cancer, with well-established minimally invasive and open techniques that also involve urinary diversion. While minimally invasive techniques, including intracorporeal diversion expectedly improve peri-operative outcomes, outcomes from either approach share similar QOL outcomes.^267–270^ Continent diversion may improve sexual function.^271–273^ Neurovascular bundle,^271^ prostate,^274^ prostate capsule,^275^ and seminal vesicle sparing approaches have promising early data for males.^276^ Female pelvic organ sparing techniques are also evolving, with early data demonstrating functional improvement.^277^ As with any organ sparing technique, careful patient selection and mature data are needed to ensure oncologic equivalence.

###### Breast

In BC surgery, image-guidance with ultrasound as compared to palpation in breast-conservation improves cosmetic and sexual outcomes,^278^ via the reducing in resection margin and positive margin status. Similarly, robotic nipple sparing mastectomy improves both surgical complications and QOL, including sexual well-being compared with open surgery,^279^ and nipple-sparing mastectomy is generally associated with higher scores for sexual well-being as compared to skin sparing.^280^

###### Gynecologic

Minimally invasive laparoscopic or robotic approaches are the most common surgical approaches to hysterectomy. Nerve-sparing surgeries remain under investigation with early data mixed.^281^ Few developments have emerged in the previous 10 years. Notably, in ovarian cancer patients, lymphadenectomy did not have impacts on sexual function other than for orgasm, showing deterioration from baseline to 12 months as compared to no lympadenectomy^282^ prompting exploration into plexus-sparing to improve functional outcomes.

###### Prostate

Radical prostatectomy minimally invasive surgical techniques incorporating nerve-sparing when feasible continue to evolve to maximize tumor control while minimizing postoperative side effects.^283^ Randomized data demonstrate that robotic assistance and nerve-sparing techniques offer improved peri-operative outcomes,^284–289^ do not offer a clear benefit to preservation of sexual function over standard approaches,^289–293^ although meta-analysis suggest some improvements.^294^ Careful consideration for well-studied techniques,^294^ and surgical experience^295^ are critical, given the risk for positive surgical margins^292^^,^^296^ and longer follow-up is necessary.^284^ Extended PLND, when indicated, does not appear to impact sexual function.^297^

###### Rectum

Surgical approaches to rectal cancer have continued to evolve with several new developments related to sexual function preservation when such options can be oncologically considered. Open and laparoscopic techniques show similar results for sexual functioning, though open techniques may have fewer sexual complications in the short term (eg, 3 months^298^); robotic techniques appear to either improve SD compared to laparoscopy^299–301^ or have similar outcomes.^300^^,^^302^ In males, several studies have investigated nerve-sparing approaches, including preservation of Denonvillier’s fascia in Total Mesorectal Excision (TME)^303^^,^^304^ resulting in lower rates of erectile and ejaculation dysfunction.

Trans anal mesorectal excision appears to improve sexual function compared to conventional laparoscopic low anterior resection,^305^ as well as vascular ligation techniques to spare the inferior mesenteric artery and pelvic autonomic nerves.^306^^,^^307^ Finally, incorporating trans-abdominal levator transection into APR improves sexual problems, particularly in males.^308^

##### Radiation interventions

###### Anal/rectal

Late sexual function outcomes from anal cancer Intensity-modulated radiation therapy (IMRT) trials demonstrated that sexual function was largely rare or underreported^309^^,^^310^ with one study showing a clinically significant worsening of dyspareunia at 5 years.^310^

As neoadjuvant rectal treatment paradigms evolve, understanding the differences in sexual outcomes with short versus long course radiotherapy will continue to be important. When comparing short to long course chemoradiation, while few female patients completed questionnaires, men experienced a decline in function, enjoyment, and sexual problems after surgery that did not return to baseline.^311^

Functional organ sparing radiotherapy techniques are emerging in anal and rectal cancer, including for females.^312–314^

###### Gynecologic

The EMBRACE group published robust data in gynecologic cancer for vaginal dose and toxicity after chemoradiotherapy and image-guided brachytherapy. Dose–effect relationships were established for Grade 2 or higher vaginal stenosis and the Posterior-Inferior Border of Symphysis points and recto-vaginal reference (RV-RP) point doses.^315^ The recommendation of keeping the external beam dose to 45 Gy and the RV-RP to <65 Gy provides actionable planning objectives to limit vaginal toxicity.^316^ Long-term data from the PORTEC-2 trial compared sexual outcomes in stage I high-intermediate-risk EC randomized to External Beam Radiation Therapy (EBRT) or vaginal brachytherapy (VBT).^317^ Sexual function largely did not differ in sexual activity or interest, vaginal dryness/shortening/pain; however, sex was less likely to be enjoyable in patients receiving VBT.

###### Penile

Recent data help to establish brachytherapy as an organ-sparing treatment for early-stage penile cancer. Extensive experience from a French center suggests that brachytherapy can provide local control rate of 82%^318^ with successful surgical salvage options for local recurrence (77.4%). Another recently published series showed that total or partial penile preservation was maintained in 87.9% at 5 years^319^ with interstitial brachytherapy. Pulsed dose rate brachytherapy also maintained penile preservation with no salvage surgery required, though 30% had some tissue necrosis.^320^

###### Prostate

Robust recent literature in prostate cancer has demonstrated similar oncologic and sexual functioning outcomes with respect to hypofractionation,^321–326^ stereotactic body radiation (SBRT),^327–332^ and dose escalation/boost^333–342^ even with inclusion of pelvic lymph nodes,^343^ providing shorter treatment options to patients.

Sexual function-sparing approaches are now possible with advanced image-guided radiotherapy. A vessel-sparing phase 2 trial of localized prostate cancer reported 5-year results that showed significant improvements in erectile function at 2 years (78% CI 71%-85%) compared to conventional (42% CI 38%-45%) or nerve-sparing prostatectomy (24% CI 22%-27%), while maintaining tumor control. Similar prospective early data support MR image-guidance^327^ to improve sexual and other QOL outcomes.^344^

Rectal spacers may also improve sexual function with SBRT/IMRT.^327^^,^^345^ In a phase 3 trial, a hydrogel spacer decreased dose to the penile bulb, associated with improvements in erectile function compared to controls.^345^

##### Systemic therapy interventions

###### Nasopharyngeal

An emerging area of interest is the impact of head and neck cancer treatment on sexual function. Recent data from a phase 3 trial of induction chemotherapy for nasopharyngeal carcinoma^346^ showed improved oncologic outcomes, with concomitant improved QOL outcomes, including sexuality, in the induction group compared to no induction. As with other disease paradigms, downstaging likely contributes to optimizing definitive treatment such that there is less associated toxicity.

###### Rectum

With TNT as a preferred treatment paradigm in certain locally advanced rectal cancers based on recent practice changing trials,^347^^,^^348^ the impacts of oxaliplatin-based neoadjuvant chemotherapy Health-Related Quality of Life (HRQOL) and sexual function are of increasing importance. Unlike other HRQOL side effects experienced with NAC, the PRODIGE 23 trial found that that surgical intervention increased ED to a lesser degree in the NAC group (31%-51%) compared to the standard of care group (42%-79%) likely due to improved downstaging facilitating surgical resection. Sexual interest was generally low, especially among women, and men with a stoma postoperatively.^349^ In TNT with CAPEOX, the EXPERT-C trial demonstrated a similar pattern with worsening QOL due to NAC, but improved sexual functioning after surgery in the NAC group.^350^ This highlights the importance of downstaging for optimizing sexual functioning outcomes.

###### Uterine

Data from the PORTEC-3 trial comparing adjuvant chemoradiation to radiation alone for high-risk endometrial cancer^351^ demonstrated that there were no substantial differences in sexual activity after 3 and 5 years, with ~34% sexually active. Sexual activity scores were significantly lower than age-matched norms, with some reporting pain and vaginal dryness.

##### Treatment choice

###### Bladder

Bladder preservation with trimodality therapy remains an alternative option to cystectomy for select patients^352^ with very favorable late QOL, including sexual QOL outcomes, at 10 years in addition to favorable oncologic outcomes.

###### Penile

Penile preservation with brachytherapy is discussed above.

###### Prostate

Recent landmark trials for localized prostate cancer have transformed shared decision making. In the ProtecT trial, which demonstrated low prostate cancer specific mortality for active monitoring, surgery and radiotherapy, active monitoring maintained the best urinary and sexual function, though this declines with age. Surgery showed the greatest impacts on sexual function and urinary leakage, while radiotherapy also had decreased sexual and bowel function compared to active monitoring.^353–356^ Men can now make an informed decision regarding the reduced disease progression and metastases and each treatments side-effect profile. Active surveillance maintains the best erectile and SD profile in those with lower risk disease.^357^^,^^358^ For high and intermediate risk prostate cancer, the benefit of dose escalation with brachytherapy compared to external beam does not confer a greater decrease in sexual function.^359^

Unfortunately, these landmark trials do not include certain aspects of sexual functioning for sexual minority men, namely receptive anal intercourse, thus treatment choice remains less well informed.^360^

Several early phase trials evaluating new approaches to localized interventions (including laser ablation, microwave ablation, and cryoablation) have been completed in recent years, though local recurrence rates are sometimes higher so careful selection and target criteria continue to be developed.^361–367^

###### Rectum

Rectal cancer management has shifted in recent years towards total neoadjuvant therapy for many patients with locally advanced disease facilitating sphincter-sparing surgery.^347^ Emerging data support non-operative management in select patients with complete clinical response^368^ offering patients an option to avoid surgical toxicities, including the SDs described above. Overall, greater sexual toxicities are experienced in patients undergoing Low anterior resection (LAR) or non-TME surgical approaches. Response-guided use of chemoradiation after neoadjuvant chemo in select patients warrants consideration based on the PROSPECT data, offering patients an option to avoid radiation toxicities,^369^ though longer follow up is needed. Careful multidisciplinary discussion and shared-decision making may provide optimal treatment selection to spare or preserve pelvic organs and enable function-sparing surgery.

## Conclusions

Section A: Sexual desire and sexual function are important components of QOL, being linked with overall health and relationship satisfaction. Numerous studies suggest that risk factors, physical inactivity, obesity, metabolic syndrome, diabetes, and CVD lead to low sexual desire and SD. Sexual problems may be a warning sign of underlying disease or a consequence of chronic diseases. Low sexual desire and SD are often associated with low testosterone levels and higher fat mass. Therefore, the prevalence of sexual problems in healthy middle-aged individuals may be associated with the presence of a portfolio of these risk factors and provide an opportunity to modify them as well as treating SD.

Section B: Regardless of the type of diagnosis under the CP umbrella, sexuality is an area that is strongly impacted in patients. Some diagnoses emphasize specificities with respect to the areas of sexual response most involved; however, the unanimous picture drawn is a wounded sexuality, with disruptive effects on romantic and social relationships, QOL, and which still too often remains unheeded by health care providers. In this sense, although early evidence is emerging of awareness and integration of sexological aspects in CP care, these interventions still too often remain limited and sporadic, far from being adopted in standard CP care practices.

Hence, it is crucial to highlight the importance of addressing two fundamental aspects: firstly, the need to assess the occurrence of sexual difficulties within the diagnostic or routine care framework of CP patients. Secondly, the emphasis should be on mitigating symptoms associated with CP and promoting sexual satisfaction rather than solely concentrating on functioning. This affects both research and care, facilitating a transition from a purely performance-oriented approach cantered on sexual function to one that prioritizes overall well-being and QOL.

Section C: The importance of managing GSM is essential for sexual recovery for the BCP and while moisturizers and lubricants have been the primary treatment, the use of alternative therapeutics including hormonal interventions are emerging and gathering significant and important data to support their use. Hormonal interventions for vaginal dryness and dyspareunia should be tailored to the patients’ specific needs and in consultation with her oncological team members. Novel, often costly device interventions and medical spas strongly advocate their use, should be viewed with cautious optimism and preference should be advocated to implement these device-based interventions in a clinical trial setting. Further data are necessary. The management of BC and GSM should include a multifaceted treatment paradigm. Precision medicine should tailor interventions, with patients’ symptoms, and her (and her oncological teams) assessment of the risk, benefit ratio in a shared decision process.

Section D: Sexual problems for women who have been diagnosed with and treated for gynecologic cancers are common, multi-faceted, and distressing, with pelvic floor dysfunctions and associated symptoms (eg, vaginal dryness, stenosis, and pain with vaginal penetration) among the most common sexual problems seen. Relative to BC, research on interventions addressing sexual problems in women with gynecologic cancer has been more limited. At present, there is a robust evidence base for the use of pelvic floor therapy and related management strategies (eg, dilator use) as well as psychosocial and behavioral interventions to treat sexual problems in women with gynecologic cancer. Well-designed controlled trials are needed to evaluate these management strategies and ensure that women experiencing SD after treatment for gynecologic cancer can obtain effective care and maintain their sexual health and well-being.

Section E: Cancer directed therapies continue to evolve to improve the therapeutic ratio and decrease toxicities such as those related to sexual function. Substantial research is dedicated to preserving sexual function are in males—a more equitable approach and distribution of resources is needed moving forward to serve female and sexual/gender minority patients.

## References

[1] Basson R, Rees P, Wang R, Montejo AL, Incrocci L. Sexual function in chronic illness. J Sex Med. 2010;7:374–388. 10.1111/j.1743-6109.2009.01621.x 20092445

[2] Jackson G, Montorsi P, Adams MA, et al. Cardiovascular aspects of sexual medicine. J Sex Med. 2010;7(4_Part_2):1608–1626. 20388161 10.1111/j.1743-6109.2010.01779.x

[3] Sadovsky R, Basson R, Krychman M, et al. Cancer and sexual problems. J Sex Med. 2010;7:349–373. 10.1111/j.1743-6109.2009.01620.x 20092444

[4] Bennett N, Incrocci L, Baldwin D, et al. Cancer, benign gynecology, and sexual function—issues and answers. J Sex Med. 2016;13(4):519–537. 10.1016/j.jsxm.2016.01.018 27045256

[5] Hackett G, Krychman M, Baldwin D, et al. Coronary heart disease, diabetes, and sexuality in men. J Sex Med. 2016;13(6):887–904. 10.1016/j.jsxm.2016.01.023 27215685

[6] Elias S, Nandi N, Fourie S, Grover L, Newman KL. Addressing factors that impact sexual well-being and intimacy in IBD patients. Curr Gastroenterol Rep. 2025;27(1):1. 10.1007/s11894-024-00956-2 39779620 PMC11711699

[7] Jaworski M . Is sexology necessary in dermatology? Review of the most common sexual dysfunction in skin disorders. Our Dermatol Online/Nasza Dermatologia. 2017;8(3):341–347. 10.7241/ourd.20173.97

[8] Leemans C, Van den Broucke S, Jeitani C. Sexual dysfunction in patients with chronic non-genital physical disease: an umbrella review. Int J Environ Res Public Health. 2025;22(2):157. 10.3390/ijerph22020157 40003383 PMC11855788

[9] Sobel T, David P. Impact of chronic medical disease on sexual function and other conditions. Obst Gynecol Clin. 2024;51(2):323–340. 10.1016/j.ogc.2024.02.006 38777487

[10] Elliott S, Birkhauser V, Courtois F, et al. Fode M sexual and reproductive health in neurological disorders: recommendations from the Fifth International Consultation on Sexual Medicine (ICSM 2024). Sex Med Rev. 2025;13:456–470. 10.1093/sxmrev/qeaf030 40728529

[11] Luria M, Park K, Gewirtz-Meydan AG, et al. Sexuality and sexual health in older adults—recommendations from the Fifth International Consultation on Sexual Medicine (ICSM 2024). Sex Med Rev. 2026;14(1):qeaf069. 10.1093/sxmrev/qeaf069 41370314

[12] Simon JA, Nappi RE, Chedraui P, et al. Genitourinary syndrome of menopause (GSM): recommendations from the Fifth International Consultation on Sexual Medicine (ICSM 2024). Sex Med Rev. 2026;14(1):qeaf055. 10.1093/sxmrev/qeaf055 40981832

[13] Armeni E, Kopanos S, Verykouki E, et al. The severity of menopausal symptoms is associated with diabetes, and cardiometabolic risk factors in middle-aged women. Minerva Endocrinol (Torino). 2023;50:151–162. 10.23736/S2724-6507.23.03905-2 37671810

[14] Katsimardou A, Patoulias D, Zografou I, et al. The impact of metabolic syndrome components on erectile function is in patients with type 2 diabetes. Metabolites. 2023;13(5):617. 10.3390/metabo13050617 PMC10222854 37233658

[15] Parmar RS, Verma S, Neelkamal Pathak VK, Bhadoria AS. Prevalence of erectile dysfunction in type 2 diabetes mellitus (T2DM) and its predictors among diabetic men. J Family Med Prim Care. 2022;11(7):3875–3879. 10.4103/jfmpc.jfmpc_1130_21 36387626 PMC9648286

[16] Peppa M, Manta A. Sexual dysfunction in diabetic patients: the role of advanced glycation end products. Curr Diabetes Rev. 2024;20(2):e070423215531. 10.2174/1573399819666230407095522 37026501

[17] Sansone A, Mollaioli D, Ciocca G, Limoncin E, Colonnello E, Jannini EA. Sexual dysfunction in men and women with diabetes: a reflection of their complications? Curr Diabetes Rev. 2022;18(1):e030821192147. 10.2174/1573399817666210309104740 33687898

[18] Winkley K, Kristensen C, Fosbury J. Sexual health and function in women with diabetes. Diab Med. 2021;38(11):e14644. 10.1111/dme.14644 34252220

[19] Yacan L, Erol O. Evaluation of sexual function among women with or without diabetes. Sex Disabil. 2019;37:77–90. 10.1007/s11195-018-9541-0

[20] Zubin HE, Yin G, Li QQ, Zeng Q, Duan J. Diabetes mellitus causes male reproductive dysfunction: a review of the evidence and mechanisms. In Vivo. 2021;35:2503–2511. 10.21873/invivo.12531 34410936 PMC8408700

[21] Jones TH, Dobs AS, Randeva H, Moore W, Parkin JM. Leflutrozole in male obesity-associated hypogonadotropic hypogonadism: Ph 2b double-blind randomised controlled trial. Eur J Endocrinol. 2023;189(3):297–308. 10.1093/ejendo/lvad099 37579053

[22] Mostafa T, Abdel-Hamid I. Ejaculatory dysfunction in men with diabetes mellitus. World J Diabetes. 2021;35(7):2503–2511. 10.21873/invivo.12531 PMC8311479 34326948

[23] Shindel AW, Serefoglu EC, Althof S, et al. Review of recent data on disorders of ejaculation and orgasm in men: recommendations from the Fifth International Consultation on Sexual Medicine. Sex Med Rev. 2025;13:318–337. 10.1093/sxmrev/qeaf016 40192552

[24] Katsimardou A, Patoulias D, Zografou I, et al. The associations between kidney function and sexual dysfunction among males and females with type 2 diabetes mellitus. Medicina (Kaunas). 2023;59(5):969. 10.3390/medicina59050969 37241201 PMC10220764

[25] Pofi R, Fiore D, De Gaetano R, et al. Phosphodiesterase-5 inhibition preserves renal hemodynamics and function in mice with diabetic kidney disease by modulating miR-22 and BMP7. Sci Rep. 2017;7:44584. 10.1038/srep44584 28294194 PMC5353686

[26] Georgiadis G, Zisis IE, Docea AO, et al. Current concepts on the reno-protective effects of phosphodiesterase 5 inhibitors in acute kidney injury: systematic search and review. J Clin Med. 2020;9:1284. 10.3390/jcm9051284 32365529 PMC7287956

[27] Flotyńska J, Filip-Bocian N, Araszkiewicz A, Zozulińska-Ziółkiewicz D, Uruska A. Physical activity protects women with type 1 diabetes from sexual dysfunctions. J Sex Marital Ther. 2023;49:932–938. 10.1080/0092623X.2023.2224319 37317780

[28] Flotynska J, Uruska A, Michalska A, Araszkiewicz A, Zozulinska-Ziolkiewicz D. Sexual dysfunction is a more frequent problem in young women with type 1 diabetes than in healthy women. J Sex Marital Ther. 2019;45(7):643–651. 10.1080/0092623X.2019.1610121 31007158

[29] Celik S, Bal MD, Kelleci M. Comparison of sexual functions in women with and without type 1 diabetes. Rev Assoc Med Bras. 2023;69(2):216–221. 10.1590/1806-9282.20220293 36790230 PMC9983465

[30] Cichocka E, Jagusiewicz M, Gumprecht J. Sexual dysfunction in young women with type 1 diabetes. Int J Environ Res Public Health. 2020;17(12):4468. 10.3390/ijerph17124468 PMC7345995 32580278

[31] Dedemoglu S, Ince S. Sexuality perceptions and sexual care needs of patients with type 1 diabetes mellitus: a mixed methods study. Sex Disabil. 2023;41:781–804. 10.1007/s11195-023-09803-0

[32] Haugstvedt A, Jørgensen J, Strandberg RB, et al. Sexual dysfunction in women with type 1 diabetes in Norway: a cross-sectional study on the prevalence and associations with physical and psychosocial complications. Diabetic Med J Br Diabetic Assoc. 2022;39(1):e14704. 10.1111/dme.14704 34596251

[33] Hylmarova S, Stechova K, Pavlinkova G, Peknicova J, Macek M, Kvapil M. The impact of type 1 diabetes mellitus on male sexual functions and sex hormone levels. Endocr J. 2020;67(1):59–71. 10.1507/endocrj.EJ19-0280 31619592

[34] Maio A, Maiorino MI, Longo M, et al. Change in circulating levels of endothelial progenitor cells and sexual function in women with type 1 diabetes. J Clin Endocrinol Metab. 2022;107(9):e3910–e3918. 10.1210/clinem/dgac316 35583559 PMC9387708

[35] Robertson C, Lin A, Smith G, et al. The impact of externally worn diabetes technology on sexual behaviour and activity, body image, and anxiety in type 1 diabetes. J Diabetes Sci Technol. 2020;14(2):303–308. 10.1177/1932296819870541 31441324 PMC7196867

[36] Dote-Montero M, De-la-O A, Castillo MJ, Amaro-Gahete F. Predictors of sexual desire and sexual function in sedentary middle-aged adults: the role of lean mass index and S-klotho plasma levels. The FIT-AGEING study. J Sex Med. 2020;17(4):665–677. 10.1016/j.jsxm.2020.01.016 32089483

[37] Kuro-o M . The Klotho proteins in health and disease. Nat Rev Nephrol Amaro-Gahete. 2019;15:27–44. 10.1038/s41581-018-0078-3 30455427

[38] Koyama D, Sato Y, Aizawa M, et al. Soluble aKlotho as a candidate for the biomarker of aging. Biochem Biophys Res Commun. 2015;(467):1019–1025. 10.1016/j.bbrc.2015.10.018 26462468

[39] Semba RD, Cappola AR, Sun K, et al. Plasma klotho and cardiovascular disease in adults. J Am Geriatr Soc. 2011;59:1596–1601. 10.1111/j.1532-5415.2011.03558.x 21883107 PMC3486641

[40] Zhang L, Liu T. Clinical implication of alterations in serum Klotho levels in patients with type 2 diabetes mellitus and its associated complications. J Diabetes Complications. 2018;32:922–930. 10.1016/j.jdiacomp.2018.06.002 30042059

[41] Semba RD, Cappola AR, Sun K, et al. Plasma Klotho and mortality risk in older community-dwelling adults. J Gerontol Ser. 2011;66:794–800. 10.1093/gerona/glr058 PMC3143348 21474560

[42] Amaro-Gahete FJ, De-la-O A, Jurado-Fasoli L, et al. Body composition and S-Klotho plasma levels in middle-aged adults: a cross-sectional study. Rejuvenation Res. 2019;22:478–483. 10.1089/rej.2018.2092 30672377

[43] Amaro-Gahete FJ, Jurado-Fasoli L, Gutiérrez Á, Ruiz JR, Castillo MJ. Association of physical activity and fitness with S-Klotho plasma levels in middle-aged sedentary adults: the FIT-AGEING study. Maturitas. 2019;123:25–31. 10.1016/j.maturitas.2019.02.001 31027673

[44] Dote-Montero M, Amaro-Gahete FJ, Jurado-Fasoli L, Gutiérrez Á, Castillo MJ. Study of the association of DHEAS, testosterone and cortisol with S-Klotho plasma levels in healthy sedentary middle-aged adults. Exp Gerontol. 2019;121:55–61. 10.1016/j.exger.2019.03.010 30928678

[45] Amaro-Gahete FJ, Jurado-Fasoli L, Espuch-Oliver A, et al. Exercise training as S-Klotho protein stimulator in sedentary healthy adults: rationale, design, and methodology. Contemp Clin Trials Commun. 2018;11:10–19. 10.1016/j.conctc.2018.05.013 30023455 PMC6022251

[46] Dandona P, Dhindsa S, Ghanim H, Saad F. Mechanisms underlying the metabolic actions of testosterone in humans: a narrative review. Diabetes Obes Metab. 2021;23(1):18–28. 10.1111/dom.14206 32991053

[47] Groti Antonič K, Antonič B, Žuran I, Pfeifer M. Testosterone treatment longer than 1 year shows more effects on functional hypogonadism and related metabolic, vascular, diabetic and obesity parameters (results of the 2-year clinical trial). Aging Male. 2020;23(5):1442–1454. 10.1080/13685538.2020.1793132 32844712

[48] Corona G, Vena W, Pizzocaro A, Vignozzi L, Sforza A, Maggi M. Testosterone therapy in diabetes and pre-diabetes. Andrology. 2023;11(2):204–214. 10.1111/andr.13367 36542412

[49] Khodarahimi S, Mazraeh N, Rahimian Bougar M, Sheikhi S. Hypogonadism and sexual functioning in males with and without diabetes type II. Sexologies. 2022;31(2):138–144. 10.1016/j.sexol.2021.04.005

[50] Shigehara K, Konaka H, Kato Y, et al. Effect of testosterone replacement therapy on sexual function and glycemic control among hypogonadal men with type 2 diabetes mellitus. Int J Impot Res. 2019;31(1):25–30. 10.1038/s41443-018-0065-z 30135606

[51] Traish AM . Major cardiovascular disease risk in men with testosterone deficiency (hypogonadism): appraisal of short, medium and long-term testosterone therapy—a narrative review. Sex Med Rev. 2023;11(4):384–394. 10.1093/sxmrev/qead031 37587664

[52] Bhasin S, Lincoff AM, Basaria S, et al. Effects of long-term testosterone treatment on cardiovascular outcomes in men with hypogonadism: rationale and design of the TRAVERSE study. Am Heart J. 2022;245:41–50. 10.1016/j.ahj.2021.11.016 34871580

[53] Lincoff AM, Bhasin S, Flevaris P, et al. TRAVERSE Study Investigators. Cardiovascular safety of testosterone-replacement therapy. N Engl J Med. 2023;389(2):107–117. 10.1056/NEJMoa2215025 37326322

[54] Botlani Esfahani S, Pal S. Does metabolic syndrome impair sexual functioning in adults with overweight and obesity? Int J Sex Health. 2019;31(2):170–185. 10.1080/19317611.2019.1611688

[55] Brettle H, Tran V, Drummond GR, et al. Sex hormones, intestinal inflammation, and the gut microbiome: major influencers of the sexual dimorphisms in obesity. Front Immunol. 2022;13:971048. 10.3389/fimmu.2022.971048 36248832 PMC9554749

[56] Deniz A, Okuyucu M. The impact of obesity on fertility and sexual function in women of childbearing age. J Obstet Gynaecol. 2022;42(7):3129–3133. 10.1080/01443615.2022.2106828 35934937

[57] Ferrández Infante A, Novella Arribas B, Khan KS, et al. Obesity and female sexual dysfunctions: a systematic review of prevalence with meta-analysis. Semergen. 2023;49(7):102022. 10.1016/j.semerg.2023.102022 37331210

[58] Fricke J, Sironi M. Sexual fluidity and BMI, obesity, and physical activity. SSM Popul Health. 2021;11:100620. 10.1016/j.ssmph.2020.100620 PMC7330605 32637556

[59] Fuchs A, Dulska A, Drosdzol-Cop A. Is weight just a number? Relationship between overweight, obesity and domains of sexual functioning among young women. Ginekol Pol. 2020;91(10):595–599. 10.5603/GP.2020.0162 33184827

[60] Ghaderpour S, Ghiasi R, Heydari H, Keyhanmanesh R. The relation between obesity, kisspeptin, leptin, and male fertility. Horm Mol Biol Clin Invest. 2021;43(2):235–247. 10.1515/hmbci-2021-0058 34931507

[61] de Almeida MM, Herbella FAM, de Godoy Dos Santos G, Valezi AC. Influence of gastric bypass on obese women sexual function—a prospective study. Obes Surg. 2021;31(8):3793–3798. 10.1007/s11695-021-05509-4 34106400

[62] Gao Z, Liang Y, Deng W, Qiu P, Li M, Zhou Z. Impact of bariatric surgery on female sexual function in obese patients: a meta-analysis. Obes Surg. 2020;30(1):352–364. 10.1007/s11695-019-04240-5 31664652

[63] Miñambres I, Sardà H, Urgell E, et al. Obesity surgery improves hypogonadism and sexual function in men without effects in sperm quality. J Clin Med. 2022;11(17):5126. 10.3390/jcm11175126 36079056 PMC9457146

[64] Nimbi FM, Virginia C, Cinzia DM, Michela DT, Gianfranco S, Emanuela P. The relation between sexuality and obesity: the role of psychological factors in a sample of obese men undergoing bariatric surgery. Int J Impot Res. 2022;34(2):203–214. 10.1038/s41443-020-00388-2 33328607

[65] Sarhan MD, Khattab M, Sarhan MD, MauriceKK HH. Impact of bariatric surgery on male sexual health: a prospective study. Obes Surg. 2021;31(9):4064–4069. 10.1007/s11695-021-05522-7 34169483

[66] Zhu G, Zhang Y, Dong J, et al. Association between body mass index and male sperm apoptosis and apoptosis-related factors. Diabetes Metab Syndr Obes. 2021;14:1043–1051. 10.2147/DMSO.S289923 33727837 PMC7955683

[67] Abdelsamea GA, Amr M, Tolba AMN, et al. Impact of weight loss on sexual and psychological functions and quality of life in females with sexual dysfunction: a forgotten avenue. Front Psychol. 2023;14:1090256. 10.3389/fpsyg.2023.1090256 36818091 PMC9929060

[68] Allen MS . Physical activity as an adjunct treatment for erectile dysfunction. Nat Rev Urol. 2019;16:553–562. 10.1038/s41585-019-0210-6 31239541

[69] Gerbild H, Larsen CM, Graugaard C, Areskoug JK. Physical activity to improve erectile function: a systematic review of intervention studies. Sex Med. 2018;6:75–89. 10.1016/j.esxm.2018.02.001 29661646 PMC5960035

[70] Stanton AM, Handy AB, Meston CM. The effects of exercise on sexual function in women. Sex Med Rev. 2018;6:548–557. 10.1016/j.sxmr.2018.02.004 29606554

[71] Pitta RM, Kaufmann O, Louzada ACS, et al. The association between physical activity and erectile dysfunction: a cross-sectional study in 20,789 Brazilian men. PLoS ONE. 2022;17(11):e0276963. 10.1371/journal.pone.0276963 36383526 PMC9668147

[72] Parazzini F, Artibani W, Carrieri G, Carmignani L, Voce S. Effect of body mass and physical activity at younger age on the risk of prostatic enlargement and erectile dysfunction: results from the 2018 #Controllati survey. Arch Ital Urol Androl. 2020;91(4):245–250. 10.4081/aiua.2019.4.245 31937085

[73] Boothby CA, Santana MJ, Norris CM, Campbell TS, Rabi DM. Sexual activity after acute coronary syndrome: a qualitative approach to patient and partner experiences. J Cardiovasc Nurs. 2021;36(5):E71–E79. 10.1097/JCN.0000000000000815 33852497 PMC8366597

[74] Buemann B, Uvnäs-Moberg K. Oxytocin may have a therapeutical potential against cardiovascular disease. Possible pharmaceutical and behavioral approaches. Med Hypotheses. 2020;138(109597). 10.1016/j.mehy.2020.109597 32032912

[75] Cipriani S, Simon JA. Sexual dysfunction as a harbinger of cardiovascular disease in postmenopausal women: how far are we? J Sex Med. 2022;19(9):1321–1332. 10.1016/j.jsxm.2022.06.007 35869024

[76] Yannas D, Frizza F, Vignozzi L, Corona G, Maggi M, Rastrelli G. Erectile dysfunction is a hallmark of cardiovascular disease: unavoidable matter of fact or opportunity to improve men's health? J Clin Med. 2021;10(10):221. 10.3390/jcm10102221 34065601 PMC8161068

[77] Mulloy E, Li S, Belladelli F, Del Giudice F, Glover F, Eisenberg ML. The risk of cardiovascular and cerebrovascular disease in men with a history of priapism. J Urol. 2023;209(1):253–260. 10.1097/JU.0000000000002962 36083148

[78] Lisco G, Bartolomeo N, De Tullio A, et al. Long-acting glucagon-like peptide 1 receptor agonists boost erectile function in men with type 2 diabetes mellitus complaining of erectile dysfunction: a retrospective cohort study. Andrology. 2024;12:633–642. 10.1111/andr.13519 37615353

[79] Defeudis G, Mazzilli R, Di Tommaso AM, et al. Effects of diet and antihyperglycemic drugs on erectile dysfunction: a systematic review. Andrology. 2023;11:282–294. 10.1111/andr.13192 35485604 PMC10084359

[80] Assaly R, Gorny D, Compagnie S, et al. The favorable effect of empagliflozin on erectile function in an experimental model of type 2 diabetes. J Sex Med. 2018;15(9):1224–1234. 10.1016/j.jsxm.2018.07.002 30145094

[81] Cannarella R, Condorelli RA, Leanza C, et al. Dapagliflozin improves erectile dysfunction in patients with type 2 diabetes mellitus: an open-label, non-randomized pilot study. Diabet Med. 2024;41:e15217. 10.1111/dme.15217 37669131

[82] Zamponi V, Defeudis G, Federico F, Faggiano A, Mazzilli R. Erectile dysfunction severity: the role of glycometabolic compensation and antihyperglycemic drugs. J Clin Med. 2022;11(23):7214. 10.3390/jcm11237214 36498788 PMC9740756

[83] Yuan P, Ma D, Gao X, et al. Liraglutide ameliorates erectile dysfunction *via* regulating oxidative stress, the RhoA/ROCK pathway and autophagy in diabetes mellitus. Front Pharmacol. 2020;11:1257. 10.3389/fphar.2020.01257 32903510 PMC7435068

[84] Cignarelli A, Genchi VA, D'Oria R, et al. Role of glucose-lowering medications in erectile dysfunction. J Clin Med. 2021;10(11):2501. 10.3390/jcm10112501 34198786 PMC8201035

[85] Giagulli VA, Carbone MD, Ramunni MI, et al. Adding liraglutide to lifestyle changes, metformin and testosterone therapy boosts erectile function in diabetic obese men with overt hypogonadism. Andrologia. 2015;3(6):1094–1103. 10.1111/andr.12099 26447645

[86] Huang CY, Shih CM, Tsao NW, et al. Dipeptidyl peptidase-4 inhibitor improves neovascularization by increasing circulating endothelial progenitor cells. Br J Pharmacol. 2012;167(7):1506–1519. 10.1111/j.1476-5381.2012.02102.x 22788747 PMC3514763

[87] Harmar AJ, Fahrenkrug J, Gozes I, et al. Pharmacology and functions of receptors for vasoactive intestinal peptide and pituitary adenylate cyclase-activating polypeptide: IUPHAR review. Br J Pharmacol. 2012;166(1):4–17. 10.1111/j.1476-5381.2012.01871.x 22289055 PMC3415633

[88] Gupta BP, Murad MH, Clifton MM, Prokop L, Nehra A, Kopecky SL. The effect of lifestyle modification and cardiovascular risk factor reduction on erectile dysfunction: a systematic review and meta-analysis. Arch Intern Med. 2011;171(20):1797–1803. 10.1001/archinternmed.2011.440 21911624

[89] Esposito K, Ciotola M, Giugliano F, et al. Mediterranean diet improves sexual function in women with the metabolic syndrome. Int J Impot Res. 2007;19(5):486–491. 10.1038/sj.ijir.3901555 17673936

[90] Wing RR, Rosen RC, Fava JL, et al. Effects of weight loss intervention on erectile function in older men with type 2 diabetes in the look AHEAD trial. J Sex Med. 2010;7(1):156–165. 10.1111/j.1743-6109.2009.01458.x 19694925 PMC4461030

[91] Rothberg AE, McEwen LN, Kraftson AT, et al. Impact of weight loss on waist circumference and the components of the metabolic syndrome. BMJ Open Diabetes Res Care. 2017;5(1):e000341. 10.1136/bmjdrc-2016-000341 PMC5337678 28316795

[92] Franz MJ, Boucher JL, Rutten-Ramos S, VanWormer JJ. Lifestyle weight-loss intervention outcomes in overweight and obese adults with type 2 diabetes: a systematic review and meta-analysis of randomized clinical trials. J Acad Nutr Diet. 2015;115(9):1447–1463. 10.1016/j.jand.2015.02.031 25935570

[93] Rejeski WJ, Ip EH, Bertoni AG, et al. Lifestyle change and mobility in obese adults with type 2 diabetes. N Engl J Med. 2012;366(13):1209–1217. 10.1056/NEJMoa1110294 22455415 PMC3339039

[94] Same RV, Al Rifai M, Feldman DI, et al. Prognostic value of exercise capacity among men undergoing pharmacologic treatment for erectile dysfunction: the FIT project. Clin Cardiol. 2017;40(11):1049–1054. 10.1002/clc.22768 28805967 PMC6490364

[95] Colberg SR, Sigal RJ, Yardley JE, et al. Physical activity/exercise and diabetes: a position statement of the American Diabetes Association. Diabetes Care. 2016;39(11):2065–2079. 10.2337/dc16-1728 27926890 PMC6908414

[96] Minami H, Furukawa S, Sakai T, et al. Physical activity and prevalence of erectile dysfunction in Japanese patients with type 2 diabetes mellitus: the Dogo study. J Diabetes Investig. 2018;9(1):193–198. 10.1111/jdi.12660 PMC5754534 28371446

[97] Cano Sokoloff N, Misra M, Ackerman KE. Exercise, training, and the hypothalamic-pituitary-gonadal axis in men and women. Front Horm Res. 2016;47:27–43. 10.1159/000445154 27348623 PMC7043068

[98] Sgro P, Romanelli F, Felici F, et al. Testosterone responses to standardized short-term sub-maximal and maximal endurance exercises: issues on the dynamic adaptive role of the hypothalamic-pituitary-testicular axis. J Endocrinol Investig. 2014;37(1):13–24. 10.1007/s40618-013-0006-0 24464446

[99] Hackett G, Kirby M. The increasing role of PDE5Is in men with diabetes and cardiovascular disease. Trends Urol Men's Health. 2023;14(4):2–7. 10.1002/tre.926

[100] Cohen SP, Vase L, Hooten WM. Chronic pain: an update on burden, best practices, and new advances. Lancet. 2021;397(10289):2082–2097. 10.1016/S0140-6736(21)00393-7 34062143

[101] Dahlhamer J, Lucas J, Zelaya C, et al. Prevalence of chronic pain and high-impact chronic pain among adults—United States, 2016. MMWR Morb Mortal Wkly Rep. 2018;67(36):1001–1006. 10.15585/mmwr.mm6736a2 30212442 PMC6146950

[102] Raja SN, Carr DB, Cohen M, et al. The revised International Association for the Study of Pain definition of pain: concepts, challenges, and compromises. Pain. 2020;161(9):1976–1982. 10.1097/j.pain.0000000000001939 32694387 PMC7680716

[103] Rosen CB, Heiman J, Leiblum S, et al. The female sexual function index (FSFI): a multidimensional self-report instrument for the assessment of female sexual function. J Sex Marital Ther. 2000;26(2):191–208. 10.1080/009262300278597 10782451

[104] Rosen RC, Riley A, Wagner G, Osterloh IH, Kirkpatrick J, Mishra A. The international index of erectile function (IIEF): a multidimensional scale for assessment of erectile dysfunction. Urology. 1997;49(6):822–830. 10.1016/S0090-4295(97)00238-0 9187685

[105] Aydın M, Bitkin A, İrkılata L, Yılmaz A, Moral C, Atilla MK. The effect of migraine and tension-type headaches on female sexual functions: a prospective, cross-sectional, controlled study. Turk J Urol. 2018;44(5):418–422. 10.5152/tud.2018.45228 30487044 PMC6134981

[106] Direkvand-Moghadam A, Suhrabi Z, Akbari M, Direkvand-Moghadam A. Prevalence and predictive factors of sexual dysfunction in Iranian women: univariate and multivariate logistic regression analyses. Korean J Family Med. 2016;37(5):293–298. 10.4082/kjfm.2016.37.5.293 PMC5039121 27688863

[107] Erdem İH, Ustabaşıoğlu F. Evaluation of sexual function and depression in female patients with fibromyalgia. Rev Assoc Med Bras. 2023;69:e20230180. 10.1590/1806-9282.20230180 37466600 PMC10352019

[108] Flegge LG, Barr A, Craner JR. Sexual functioning among adults with chronic pain: prevalence and association with pain-related outcomes. Pain Med. 2023;24(2):197–206. 10.1093/pm/pnac117 35929084

[109] Hayta E, Mert DG. Potential risk factors increasing the severity of sexual dysfunction in women with fibromyalgia. Sex Disabil. 2017;35(2):147–155. 10.1007/s11195-016-9472-6

[110] Jedel S, Hood MM, Keshavarzian A. Getting personal: a review of sexual functioning, body image, and their impact on quality of life in patients with inflammatory bowel disease. Inflamm Bowel Dis. 2015;21(4):923–938. 10.1097/MIB.0000000000000257 25789923 PMC4369789

[111] Kayhan F, Küçük A, Satan Y, İlgün E, Arslan Ş, İlik F. Sexual dysfunction, mood, anxiety, and personality disorders in female patients with fibromyalgia. Neuropsychiatr Dis Treat. 2016;12:349–355. 10.2147/NDT.S99160 26937190 PMC4762461

[112] Nemichandra SC, Pradeep R, Harsha S, Radhika K, Iqbal R. Erectile dysfunction in migraine in Indian patients. Ann Indian Acad Neurol. 2020;23(6):792–795. 10.4103/aian.AIAN_554_19 33688129 PMC7900724

[113] Odole AC, Olugbenga-Alfred AA. Sexual functioning and selected clinical and psychosocial factors among individuals with chronic non-specific low back pain in Ibadan, Nigeria. Sex Disabil. 2018;36(2):185–194. 10.1007/s11195-018-9522-3

[114] Perez de Arce E, Quera R, Ribeiro Barros J, Yukie Sassaki L. Sexual dysfunction in inflammatory bowel disease: what the specialist should know and ask. Int J General Med. 2021;14:2003–2015. 10.2147/IJGM.S308214 PMC8163621 34079340

[115] Pinto B, Grover S, Dhooria A, Rathi M, Sharma A. Sexual functioning and its correlates in premenopausal married Indian women with systemic lupus erythematosus. Int J Rheum Dis. 2019;22(10):1814–1819. 10.1111/1756-185X.13675 31424179

[116] Shmidt E, Suárez-Fariñas M, Mallette M, et al. Erectile dysfunction is highly prevalent in men with newly diagnosed inflammatory bowel disease. Inflamm Bowel Dis. 2019;25(8):1408–1416. 10.1093/ibd/izy401 30861068 PMC10424100

[117] Shmidt E, Suárez-Fariñas M, Mallette M, et al. A longitudinal study of sexual function in women with newly diagnosed inflammatory bowel disease. Inflamm Bowel Dis. 2019;25(7):1262–1270. 10.1093/ibd/izy397 30726913 PMC11079919

[118] Solmaz V, Ceviz A, Aksoy D, et al. Sexual dysfunction in women with migraine and tension-type headaches. Int J Impot Res. 2016;28:201–204. 10.1038/ijir.2016.22 27654031

[119] Tański W, Dudek K, Tomasiewicz A, Świątoniowska-Lonc N. Sexual dysfunction and quality of life in patients with rheumatoid arthritis. Int J Environ Res Public Health. 2022;19(5):5. 10.3390/ijerph19053088 PMC8910488 35270781

[120] Thornton KGS, Robert M. Prevalence of pelvic floor disorders in the fibromyalgia population: a systematic review. J Obstet Gynaecol Can. 2020;42(1):72–79. 10.1016/j.jogc.2019.02.013 31320239

[121] Nimbi FM, Mesce M, Limoncin E, Renzi A, Galli F. Role of sexuality in women with chronic pain: results from an Italian cross-sectional study on chronic headache, fibromyalgia, and vulvodynia. Int J Clin Health Psychol. 2024;24(2). 10.1016/j.ijchp.2024.100472 PMC11214997 38953047

[122] Eluri S, Cross RK, Martin C, et al. Inflammatory bowel diseases can adversely impact domains of sexual function such as satisfaction with sex life. Dig Dis Sci. 2018;63(6):1572–1582. 10.1007/s10620-018-5021-8 29564672 PMC5955825

[123] Mantzouranis G, Fafliora E, Glanztounis G, Christodoulou DK, Katsanos KH. Inflammatory bowel disease and sexual function in male and female patients: an update on evidence in the past ten years. J Crohn's Colitis. 2015;9(12):1160–1168. 10.1093/ecco-jcc/jjv140 26254470

[124] Coluzzi F, Billeci D, Maggi M, Corona G. Testosterone deficiency in non-cancer opioid-treated patients. J Endocrinol Investig. 2018;41(12):1377–1388. 10.1007/s40618-018-0964-3 30343356 PMC6244554

[125] Els C, Jackson TD, Kunyk D, et al. Adverse events associated with medium- and long-term use of opioids for chronic non-cancer pain: an overview of Cochrane reviews. Cochrane Database Syst Rev. 2017;10:CD012509. 10.1002/14651858.CD012509.pub2 29084357 PMC6485910

[126] Kleinmann B, Firoozabadi NK, Wolter T. A Cross-cultural perspective on intrathecal opioid therapy between German and Iranian patients. Cult Med Psychiatry. 2021;45(2):218–233. 10.1007/s11013-020-09682-6 32725439 PMC8463370

[127] Antony T, Alzaharani SY, El-Ghaiesh SH. Opioid-induced hypogonadism: pathophysiology, clinical and therapeutics review. Clin Exp Pharmacol Physiol. 2020;47(5):741–750. 10.1111/1440-1681.13246 31886562

[128] Marudhai S, Patel M, Valaiyaduppu Subas S, et al. Long-term opioids linked to hypogonadism and the role of testosterone supplementation therapy. Cureus. 2020;12:e10813. 10.7759/cureus.10813 33173622 PMC7645309

[129] Richardson E, Bedson J, Chen Y, Lacey R, Dunn K, m. Increased risk of reproductive dysfunction in women prescribed long-term opioids for musculoskeletal pain: a matched cohort study in the clinical practice research datalink. Eur J Pain. 2018;22(9):1701–1708. 10.1002/ejp.1256 29873872

[130] Ajo R, Segura A, Inda M-M, et al. Erectile dysfunction in patients with chronic pain treated with opioids. Med Clín (English Edition). 2017;149(2):49–54. 10.1016/j.medcli.2016.12.038 28236471

[131] Birke H, Ekholm O, Højsted J, Sjøgren P, Kurita GP. Chronic pain, opioid therapy, sexual desire, and satisfaction in sexual life: a population-based survey. Pain Med. 2019;20(6):1132–1140. 10.1093/pm/pny122 29982788

[132] Chou R, Turner JA, Devine EB, et al. The effectiveness and risks of long-term opioid therapy for chronic pain: a systematic review for a National Institutes of Health pathways to prevention workshop. Ann Intern Med. 2015;162(4):276–286. 10.7326/M14-2559 25581257

[133] López-Rodríguez MM, Fernández AP, Hernández-Padilla JM, Fernández-Sola C, Fernández-Medina IM, Granero-Molina J. Dyadic and solitary sexual desire in patients with fibromyalgia: a controlled study. J Sex Med. 2019;16(10):1518–1528. 10.1016/j.jsxm.2019.07.026 31501059

[134] Riediger C, Schuster T, Barlinn K, Maier S, Weitz J, Siepmann T . Adverse effects of antidepressants for chronic pain: a systematic review and meta-analysis. Front. Neurol. 2017;8:307. 10.3389/fneur.2017.00307 PMC5510574 28769859

[135] Walitt B, Urrútia G, Nishishinya MB, Cantrell SE, Häuser W. Selective serotonin reuptake inhibitors for fibromyalgia syndrome. Sao Paulo Med J. 2015;133:454–454. 10.1590/1516-3180.20151335T1 26046493 PMC4755337

[136] Granero-Molina J, Matarín Jiménez TM, Ramos Rodríguez C, Hernández-Padilla JM, Castro-Sánchez AM, Fernández-Sola C. Social support for female sexual dysfunction in fibromyalgia. Clin Nurs Res. 2018;27(3):296–314. 10.1177/1054773816676941 29421939

[137] Nimbi FM, Galizia R, Rossi R, et al. The biopsychosocial model and the sex-positive approach: an integrative perspective for sexology and general health care. Sex Res Soc Policy. 2022;19:894–908. 10.1007/s13178-021-00647-x

[138] Odabas FO . Evaluation of sexual dysfunction in female patients with tension-type headache. Istanbul Med J. 2017;18(2):58–61. 10.5152/imj.2017.06978

[139] Deyo RA, Korff MV, Duhrkoop D. Opioids for low back pain. BMJ. 2015;350:g6380. 10.1136/bmj.g6380 25561513 PMC6882374

[140] Ferrari S, Vanti C, Giagio S, et al. Low back pain and sexual disability from the patient’s perspective: a qualitative study. Disabil Rehabil. 2022;44(10):2011–2019. 10.1080/09638288.2020.1817161 32931339

[141] Matarín Jiménez TM, Fernández-Sola C, Hernández-Padilla JM, Correa Casado M, Antequera Raynal LH, Granero-Molina J. Perceptions about the sexuality of women with fibromyalgia syndrome: a phenomenological study. J Adv Nurs. 2017;73(7):1646–1656. 10.1111/jan.13262 28122137

[142] Nilsing Strid E, Ekelius-Hamping M. Experiences of sexual health in persons with hip and knee osteoarthritis: a qualitative study. BMC Musculoskelet Disord. 2020;21(1):576. 10.1186/s12891-020-03596-5 32838770 PMC7445899

[143] Mazo JPS, Estrada MG. Repercussions of chronic pain on Couple’s dynamics: perspectives of women with fibromyalgia. Revista Colombiana de Psicología. 2019;28(2):47–61. 10.15446/rcp.v28n2.71021

[144] Yılmaz R, Karpuz S, Akdere E, Yılmaz H. Evaluation of sexual dysfunction in females with neck and upper back myofascial pain syndrome: a cross-sectional study. Rheumatol Int. 2023;43(9):1723–1732. 10.1007/s00296-023-05359-6 37294458

[145] de Almeida PHTQ, de Ferreira C, Kurizky PS, Muniz LF, da Mota LMH. How the rheumatologist can guide the patient with rheumatoid arthritis on sexual function. Rev Bras Reumatol. 2015;55:458–463. 10.1016/j.rbr.2014.08.009 25794992

[146] Espinoza G, Maldonado G, Narvaez J, Guerrero R, Citera G, Rios C. Beyond rheumatoid arthritis evaluation: what are we missing? Open Access Rheumatol. 2021;13:45–55. 10.2147/OARRR.S298393 33790666 PMC8007602

[147] Edwards S, Mandeville A, Petersen K, Cambitzi J, de Williams AC, Herron K. ‘ReConnect’: a model for working with persistent pain patients on improving sexual relationships. Br J Pain. 2020;14(2):82–91. 10.1177/2049463719854972 32537146 PMC7265594

[148] Nagpal M, Jangid RK, Sathyanarayan Rao TS. A comparative study of the sexual functioning of women with primary headache in India. Indian J Psychiatry. 2018;60(2):224–228. 10.4103/psychiatry.IndianJPsychiatry_290_17 30166680 PMC6102962

[149] Briggs AM, Slater H, Van Doornum S, et al. Chronic primary or secondary noninflammatory musculoskeletal pain and disrupted sexual function and relationships: a systematic review. Arthritis Care Res. 2022;74(6):1019–1037. 10.1002/acr.24711 34057305

[150] Finn E, Morrison TG, McGuire BE. Correlates of sexual functioning and relationship satisfaction among men and women experiencing chronic pain. Pain Med. 2018;19(5):942–954. 10.1093/pm/pnx056 29741742

[151] Ragab OA, Mohamed ES, Ghazi EA, Basiouny MA. Sexual dysfunction in Egyptian patients with migraine. Egypt J Neurol Psychiatry Neurosurg. 2023;59(1):104. 10.1186/s41983-023-00709-4

[152] Santos-Iglesias P, Crump L, Henry JL, LaChapelle DL, Byers ES. The sexual lives of women living with fibromyalgia: a qualitative study. Sex Disabil. 2022;40(4):669–685. 10.1007/s11195-022-09748-w

[153] Palacios S, Hood S, Philips A, Savania N, Krychman M. A randomized trial on the effectiveness and safety of 5 water based personal lubricants. J Sex Med. 2023;20(4):498–506. 10.1093/jsxmed/qdad005 36781402

[154] World Health Organization . Use and procurement of additional lubricants for male and female condoms: WHO/UNFA/FH13. In: Advisory Norte. World Health Organization; 2012: https://iris.who.int/bitstream/handle/10665/76581/WHO_RHR_12.34_eng.pdf

[155] Hickey M, Marino J, Braat., Wong S. A randomized double-blind cross over trial comparing a silicone versus water- based lubricant for sexual discomfort after breast cancer. Breast Cancer Res Treat. 2016;158(1):79–90. 10.1007/s10549-016-3865-1 27306420

[156] Casiano EE, Hobson D, Aschkenazi S, et al. Non-Estrogen therapies for treatment of genitourinary syndrome of menopause: a systematic review. Obstet Gynecol. 2023;142(3):555–570. 10.1097/AOG.0000000000005288 37543737

[157] Seav SM, Dominick SA, Stepanyuk B, et al. Management of sexual dysfunction in breast cancer survivors: a systematic review. Women’s Midlife Health. 2015;1:1–27. 10.1186/s40695-015-0009-4 30766696 PMC6297963

[158] Biglia N, Peano E, Sgandurra P, et al. Low dose vaginal estrogen or vaginal moisturizer in breast cancer survivors with urogenital atrophy: a preliminary study. Gynecol Endocrinol. 2010;26(6):404–412. 10.3109/09513591003632258 20196634

[159] Juliato PT, Rodrigues AT, Stahlschmidt R, Juliato CRT, Mazzola PG. Can polyacrylic acid treat sexual dysfunction in women with breast cancer receiving tamoxifen. Climacteric. 2017;20(1):62–66. 10.1080/13697137.2016.1258396 27876429

[160] Advani P, Brewster AM, Baum GP, Schover L. A pilot randomized trial to prevent sexual dysfunction in postmenopausal breast survivors starting adjuvant aromatase inhibitor therapy. J Cancer Surviv. 2017;11(4):477–485. 10.1007/s11764-017-0606-3 28229275

[161] Chatsiproios D, Schmidts-Winkler IM, Konig L, Wasur C, Abels C. Topical treatment of vaginal dryness with a non-hormone cream in women undergoing breast cancer treatment—an open Prospective Multicenter Study. PLoS ONE. 2019;14(1):e0210967. 10.1371/journal.pone.0210967 30677065 PMC6345451

[162] Faubion S, Larkin L, Stuenkel C, et al. Consensus recommendation: management of genitourinary syndrome of menopause in women with or at high risk for breast cancer: consensus recommendations from the North American Menopause Society and the International Society for the Study of Womens Sexual Health. Menopause. 2018;25(6):596–608. 10.1097/GME.0000000000001121 29762200

[163] Treatment of Urogenital Symptoms in Individuals With a History of Estrogen-dependent Breast CancerClinical Consensus. Obstetrics & Gynecology. American College of Obstetricians and Gynecologists (ACOG) Publications. Clinical consensus. Treatment of urogenital symptoms win individuals with a history of estrogen dependent breast cancer. 2021;138:950–958. 10.1097/AOG.0000000000004601 34794166

[164] Runowicz CD, Leach CR, Henry NL, et al. American Cancer Society/American Society of Clinical Oncology Breast Cancer Survivorship Care Guideline. CA Cancer J Clin. 2016;66(1):43–73. 10.3322/caac.21319 26641959

[165] Carter J. Lcchetti C., Anderson BL., Barton Dl., Bolte S Damast S et al.(2018) Interventions to address sexual problems in people with cancer. American Society of Clinical Oncology Clinical Practice Guideline Adaptation of Cancer Care Ontario Guideline J Clic Oncol 36: (5) 492–511. 10.1200/JCO.2017.75.89 29227723

[166] Perez-Lopez FR, Philips N, Viera-Baptista P, Cohen-Sacher B, Fialho S, Stockdale C. Management of postmenopausal vulvovaginal atrophy: recommendations of the International Society for the Study of Vulvovaginal Disease. Gynecol Endocrinol. 2021;37(8):746–752. 10.1080/09513590.2021.1943346 34169794

[167] The menopause society position statement: the 2020 genitourinary syndrome of menopause position statement of the North American Menopause Society. Menopause. 2020;27(9):976–992. 10.1097/GME.0000000000001609 32852449

[168] Lopez D . Management of Genitourinary Syndrome of menopause in breast cancer survivors: an update. World. J Clin Oncol. 2022;13:71. 10.5306/wjco.v13.i2.71 PMC8894268 35316932

[169] Shim S, Min PK, Chung YJ, Kim MR. Updates on therapeutic alternatives for genitourinary syndrome of menopause: hormonal and non-hormonal management. J Menopausal Med. 2021;27:1–7. 10.6118/jmm.20034 33942583 PMC8102810

[170] Sussman TA, Kruse ML, Thacker HL, Abraham J. Management of Genitourinary Urinary Syndrome of menopause in breast cancer survivors receiving endocrine therapy. J Oncol Pract. 2019;15(7):363–370. 10.1200/JOP.18.0071 31291563

[171] Kagan R, Kellogg- SS, Parish S. Practical treatment considerations in the management of Genitourinary Syndrome of menopause. Drugs Aging. 2019;36(10):897–908. 10.1007/s40266-019-00700-w 31452067 PMC6764929

[172] Zuo SW, Wu H, Shen W. Vaginal estrogen and mammogram results: case series and review of literature on treatment of genitourinary syndrome of menopause (GSM) in breast cancer survivors. Menopause. 2018;25(7):828–836. 10.1097/GME.0000000000001079 29533365

[173] Sassarini J, Perera M, Spowart K, et al. Managing vulvovaginal atrophy after breast cancer. Post Reproductive Health. 2018;24(4):163–165. 10.1177/2053369118805344 30348046

[174] Moreno AC, Sikka SK, Thacker HL. Genitourinary syndrome of menopause in breast cancer survivors: treatments are available. Cleve Clin J Med. 2018;85:760–766. 10.3949/ccjm.85a.17108 30289755

[175] Biglia N, Bounous VE, Sgro LG, D’Alonzo MD, Pecchio S, Nappi RE. Genitourinary syndrome of menopause in brest cancer survivors: are we facing new and safe hopes. Clin Breast Cancer. 2015;15(6):413–420. 10.1016/j.clbc.2015.06.005 26198332

[176] Pinto AC . Sexuality and brest cancer: prime time for young patients. J Thorc Dis. 2013;5(S1):S81–S86. 10.3978/j.issn2072-1439.2013.05.23 PMC3695536 23819031

[177] Mension E, Alonso I, Castelo-Braco. Genitourinary syndrome of menopause: current treatment options in breast cancer survivors—systematic review. Maturitas. 2020;143:47–58. 10.1016/j.mauritas.2020.08.010 33308636

[178] Agarwal P, Singh SM, Able C, Dumas K, et al. Safety of vaginal estrogen therapy for genitourinary syndrome of menopause in women with a history of breast cancer. Obstet Gynecol. 2023;142(3):660–668. 10.1097/AOG.0000000000005294 37535961

[179] Mension E, Alonso I, Cebrecos I, et al. Safety of Prasterone in breast cancer survivors treated with aromatase inhibitors: the Vibra Pilot Study. Climacteric. 2022;25(5):476–482. 10.1080/13697137.2022.2050208 35343852

[180] Cold S, Cold F, Jensen MB, Cronin-Fenton D, et al. Systemic or vaginal hormone therapy after early breast cancer: a Danish observational cohort study. JNCI J Natl Cancer Inst. 2022;114(10):1347–1354. 10.1093/jnci/djac112 35854422 PMC9552278

[181] Cucinella L, Tiranini L, Cassani C, Martella S, Nappi RE. Genitourinary syndrome of menopause in breast cancer survivors: current perspectives on the role of laser therapy. Int J Women's Health. 2023;15(15):1261–1282. 10.2147/IJWH.S414509 37576184 PMC10422970

[182] Okui N . Vaginal laser treatment for genitourinary syndrome of menopause in breast cancer survivors: a narrative review. Cureus. 2023;15(9):e45495. 10.7759/cureus.45495 37731685 PMC10508706

[183] D’Oria O, Giannini A, Buzzaccarini G, Tinelli A, et al. Fractional Co2 laser for vuvlovaginal atrophy in Gynecology cancer patients: a valid therapeutic choice: a systematic review. Eur J Obstet Gynecol Reprod Biol. 2022;277:84–89. 10.1016/j.ejogrb.2022.08.012 36037664

[184] Filippini M, Porcari I, Ruffolo AF, Casiraghi A, et al. CO2 laser therapy and genitourinary syndrome of menopause. A systematic review and meta analysis. J Sex Med. 2022;19(3):452–470. 10.1016/j.jsxm.2021.12.010 35101378

[185] Tranoulis A, Georgiou D, Michala L. Laser treatment for the management of Genitourinary Syndrome of menopause after breast cancer hope or hype. Int Urogynecol J. 2019;30:1879–1880. 10.1007/s00192-019-04051-3 31321465

[186] Jha S, Wyld L, Krishnaswamy PH. The impact of vaginal laser treatment for genitourinary syndrome of menopause in breast cancer survivors: a systematic review and meta-analysis. Clin Breast Cancer. 2019;19(4):e556–e562. 10.1016/j.clbc.2019.04.007 31227415

[187] Knight C, Logan V, Fenion D. A systematic review of laser therapy for vulvovaginal atrophy/genitourinary syndrome of menopause in breast cancer survivors. E cancer. 2019;13:998. 10.3332/ecancer.2019.988 PMC6974376 32010212

[188] Pitsouni E, Grigoriadis T, Falagas ME, Salvatore S, Athanasiou S. Laser therapy for the Genitourinaary syndrome of menopause: a systematic review and meta-analysis. Maturitas. 2017;103:78–88. 10.1016/j.maturitas.2017.06.029 28778337

[189] Salvatore S, Pitsouni E, Del Deo F, Parma M, Athanasiou S, et al. Sexual function in women suffering from genitourinary syndrome of menopause treated with fractional laser. Sexual Medicine Rev. 2017;5(4):486–494. 10.1016/j.sxmr.2017.07.003 28843942

[190] Benini V, Ruffolo AF, Casiraghi A, et al. New innovations for the treatment of VulvoVaginal atrophy: an update review. Medicina. 2022;58:770. 10.3390/medicina58060770 35744033 PMC9230595

[191] Mension E, Alonso I, Angles-Acedo S, et al. Effect of fractional carbon dioxide versus sham laser on sexual function in survivors of breast cancer on receiving aromatase inhibitors for genitourinary syndrome of menopause: the LIGHT randomized Clinical Trail. JAMA Netw. 2023;6(2):e2255697. 10.1001/jamanetworkopen.2022.55697 PMC9918877 36763359

[192] Gold D, Nicolay L, Avian A, Greimel E, Balic M, et al. Vaginal laser therapy versus hyaluronic acid suppositories for women with symptoms of urogenital atrophy after the treatment for breast cancer. A Randomized Controlled Trial. Maturitas. 2023;167:1–7. 10.1016/j.maturitas.2022.08.013 36279690

[193] Flint R, Cardozo L, Grigoriadis T, Rantell A, Pitsouni E, et al. Rational and design for fractional micro ablative CO2 laser versus photothermal non-ablative erbium: YAG laser for the Management of Genitourinary Syndrome of menopause: a non-inferiority, single blind randomized controlled trial. Climacteric. 2019;22(93):307–311. 10.1080/13697137.2018.1559806 30676818

[194] Areas F, Valadares ALR, Conde DM, Costa-Paiva L. The effects of vaginal erbium laser treatment on sexual function and vaginal health in women with a history of breast cancer and symptoms of genitourinary syndrome of menopause: a prospective study. Menopause. 2019;26(9):1052–1058. 10.1097/GME.0000000000001353 31453969

[195] Arab M, Vasef M, Talayeh M, Hosseini MS, Farzaneh F. The effect of radiofrequency therapy on sexual function in female caner survivors (gynecologic and breast) a non- cancer menopausal women: a single arm trial. J Laser Med Sci. 2023;14(14):e32. 10.34172/jlms.2023.32 PMC10517569 37744017

[196] Athanasiou S, Pitsouni E, Douskos A, Salvadore S, Loutradidis D, et al. Intravaginal energy based devices and sexual health of female cancer survivors: a systematic review and meta-analysis. Lasers Med Sci. 2020;35:1–11. 10.1007/s10103-019-02855-9 31396795

[197] Sarmento AC, Fernandes FS, Maconi C, Giraldo C, et al. Impact of micro ablative fractional radiofrequency on the vaginal health, microbiota, and cellularity of postmenopausal women. Clinics (San Paulo). 2020, 2020;75:e1750. 10.6061/clinics/2020/e1750 PMC7384205 32756817

[198] Benini V, Ruffolo AF, Casiraggi A, Degliuomini RS, Frigerio M, et al. New innovations for the treatment of vulvovaginal atrophy an UpToDate review. Medicina. 2022;58(6):770. 10.3390/medicina58060770 35744033 PMC9230595

[199] Donovitz G, Cotten M. Breast cancer incidence reduction in women treated with subcutaneous testosterone: testosterone therapy and breast cancer incidence study. Eur J Breast Health. 2021;17(2):150–156. 10.4274/ejbh.galenos.2021.6213 33870115 PMC8025725

[200] Glaser R, Dimitrakakis C. Testosterone implant therapy in women with and without breast cancer: rationale, experience, evidence. Androgens Clin Res Ther. 2021;2(1):94–109. 10.1089/andro.2021.0003

[201] Mendoza N, Carrion R, Mendoza-Huertas L, Jurado AR. Efficacy and safety of treatments to improve dyspareunia in breast cancer survivors: a systematic review. Breast Care. 2020;15:599–607. 10.1159/000506148 33447234 PMC7768121

[202] Melisko ME, Goldman ME, Hwang J, et al. Vaginal testosterone cream versus estradiol vaginal ring for dryness or decreased libido in women receiving an aromatase inhibitor for early-stage breast cancer: a randomized clinical trial. JAMA Oncol. 2017;3(3):313–319. 10.1001/jamaoncol.2016.3904 27832260

[203] Lemke EA, Madsen LT, Dains JE. Vaginal testosterone for management of aromatase inhibitor related sexual dysfunction: an integrative review. Oncol Nurse Forum. 2017;44:296–301. 10.1188/17.ONF.296-301 28635978

[204] Hummel SB, Van Lankveld J, Oldenburg H, et al. Efficacy of internet based cognitive Behavioral therapy in improving sexual functioning of breast cancer survivors: results of a randomized controlled trial. J Clin Oncol. 2017;35(12):1328–1340. 10.1200/JCO.2016.69.6021 28240966

[205] Gorman JR, Drizin JH, Smith E, Corey S, Temple M, Rendle KA. Feasiblity of mindful after cancer: pilot study of a virtual mindfulness-based intervention for sexual health in cancer survivorship. J Sex Med. 2022;19(7):1131–1146. 10.1016/j.jsxm,2022.03.618 35523716

[206] American Cancer Society . Cancer Facts & Figures 2024. Atlanta: American Cancer Society; 2024.

[207] Cianci S, Tarascio M, Arcieri M, et al. Post treatment sexual function and quality of life of patients affected by cervical cancer: a systematic review. Medicina (Kaunas). 2023;59(4):704. 10.3390/medicina59040704 PubMed PMID: 37109662; PMCID: PMC10144819 PMC10144819 37109662

[208] Malandrone F, Bevilacqua F, Merola M, et al. The impact of vulvar cancer on psychosocial and sexual functioning: a literature review. Cancers (Basel). 2021;14(1):63. 10.3390/cancers14010063 PubMed PMID: 35008225; PMCID: PMC8750175 PMC8750175 35008225

[209] Qian M, Wang L, Xing J, Shan X, Wu J, Liu X. Prevalence of sexual dysfunction in women with cervical cancer: a systematic review and meta-analysis. Psychol Health Med. 2023;28(2):494–508. 10.1080/13548506.2022.2110270 35946648

[210] Tramacere F, Lancellotta V, Casa C, et al. Assessment of sexual dysfunction in cervical cancer patients after different treatment modality: a systematic review. Medicina (Kaunas). 2022;58(9):1223. 10.3390/medicina58091223 PubMed PMID: 36143900; PMCID: PMC9504584 PMC9504584 36143900

[211] Chang CP, Wilson CM, Rowe K, et al. Sexual dysfunction among gynecologic cancer survivors in a population-based cohort study. Support Care Cancer. 2022;31(1):51. 10.1007/s00520-022-07469-6 PubMed PMID: 36526929; PMCID: PMC9850804 36526929 PMC9850804

[212] Lin H, Fu H-C, Wu C-H, et al. Evaluation of sexual dysfunction in gynecologic cancer survivors using DSM-5 diagnostic criteria. BMC Womens Health. 2022;22(1):1. 10.1186/s12905-021-01559-z 34986812 PMC8734329

[213] Kulkarni A, Sun G, Manuppelli S, et al. Sexual health and function among patients receiving systemic therapy for primary gynecologic cancers. Gynecol Oncol. 2022;165(2):323–329. 10.1016/j.ygyno.2022.03.008 PubMed PMID: 35307202 35307202

[214] Xue Z, Zhu X, Teng Y. Comparison of nerve-sparing radical hysterectomy and radical hysterectomy: a systematic review and meta-analysis. Cell Physiol Biochem. 2016;38(5):1841–1850. 10.1159/000443122 PubMed PMID: 27160267 27160267

[215] Novackova M, Pastor Z, Chmel R Jr, Mala I, Chmel R. Sexuality and quality of life after nerve-sparing radical hysterectomy for cervical cancer: a prospective study. Taiwan J Obstet Gynecol. 2022;61(4):641–645. 10.1016/j.tjog.2021.10.006 PubMed PMID: 35779914 35779914

[216] 349 Shankar A, Patil J, Luther A, et al. Sexual dysfunction in carcinoma cervix: assessment in post treated cases by LENTSOMA scale. Asian Pac J Cancer Prev. 2020;21(2):349–354. 10.31557/apjcp.2020.21.2.349 PubMed PMID: 32102510; PMCID: PMC7332127 PMC7332127 32102510

[217] Roussin M, Lowe J, Hamilton A, Martin L. Factors of sexual quality of life in gynaecological cancers: a systematic literature review. Arch Gynecol Obstet. 2021;304(3):791–805. 10.1007/s00404-021-06056-0 PubMed PMID: 33847794; PMCID: PMC8325662 33847794 PMC8325662

[218] Harris MG . Sexuality and menopause: unique issues in gynecologic cancer. Semin Oncol Nurs. 2019;35(2):211–216. 10.1016/j.soncn.2019.02.008 30871843

[219] Logue CA, Pugh J, Foden P, et al. Psychosexual morbidity in women with ovarian cancer: evaluation by germline BRCA gene mutational status. Sex Med. 2022;10:100465. 10.1016/j.esxm.2021.100465 PubMed PMID: 34922303; PMCID: PMC8847828 PMC8847828 34922303

[220] American Psychiatric Association . Diagnostic and Statistical Manual of Mental Disorders:DSM-5. 5th ed; 2013. Washington, D.C.: American Psychiatric Publishing.

[221] Oxley S, Xiong R, Wei X, et al. Quality of life after risk-reducing hysterectomy for endometrial cancer prevention: a systematic review. Cancers (Basel). 2022;14(23):5832. 10.3390/cancers14235832 PubMed PMID: 36497314; PMCID: PMC9736914 PMC9736914 36497314

[222] Wei X, Oxley S, Sideris M, et al. Quality of life after risk-reducing surgery for breast and ovarian cancer prevention: a systematic review and meta-analysis. Am J Obstet Gynecol. 2023;229(4):388–409 e4. 10.1016/j.ajog.2023.03.045 PubMed PMID: 37059410 37059410

[223] Bradford A, Fellman B, Urbauer D, et al. Assessment of sexual activity and dysfunction in medically underserved women with gynecologic cancers. Gynecol Oncol. 2015;139(1):134–140. 10.1016/j.ygyno.2015.08.019 26325527 PMC4587341

[224] Arthur EK, Bissram J, Rechenberg K, et al. Sexual health, and intimacy after cancer treatment in women of color: a systematic review. Psychooncology. 2022;31(10):1637–1650. 10.1002/pon.6005 35852026

[225] Arthur EK, Worly B, Carpenter KM, et al. Let's get it on: addressing sex and intimacy in older cancer survivors. J Geriatr Oncol. 2021;12(2):312–315. 10.1016/j.jgo.2020.08.003 32912736

[226] Serçekuş Ak P, Partlak Günüşen N, Göral Türkcü S, Özkan S. Sexuality in Muslim women with gynecological cancer. Cancer Nurs. 2020;43(1):E47–E53. 10.1097/NCC.0000000000000667 31805026

[227] Haylen BT, de Ridder D, Freeman RM, et al. An International Urogynecological Association (IUGA)/International Continence Society (ICS) joint report on the terminology for female pelvic floor dysfunction. Neurourol Urodyn. 2010;29(1):4–20. 10.1002/nau.20798 19941278

[228] Ramaseshan AS, Felton J, Roque D, Rao G, Shipper AG, Sanses TVD. Pelvic floor disorders in women with gynecologic malignancies: a systematic review. Int Urogynecol J. 2018;29(4):459–476. 10.1007/s00192-017-3467-4 PubMed PMID: 28929201; PMCID: PMC7329191 28929201 PMC7329191

[229] Barcellini A, Dominoni M, Dal Mas F, et al. Sexual health dysfunction after radiotherapy for Gynecological cancer: role of physical rehabilitation including pelvic floor muscle training. Front Med. 2022;8:8. 10.3389/fmed.2021.813352 PMC8852813 35186978

[230] Shan X, Qian M, Wang L, Liu X. Prevalence of pelvic floor dysfunction and sexual dysfunction in cervical cancer survivors: a systematic review and meta-analysis. Int Urogynecol J. 2023;34(3):655–664. 10.1007/s00192-022-05326-y PubMed PMID: 36001098 36001098

[231] Hofsjo A, Bergmark K, Blomgren B, Jahren H, Bohm-Starke N. Radiotherapy for cervical cancer—impact on the vaginal epithelium and sexual function. Acta Oncol. 2018;57(3):338–345. 10.1080/0284186X.2017.1400684 29140150

[232] Chapman CH, Heath G, Fairchild P, et al. Gynecologic radiation oncology patients report unmet needs regarding sexual health communication with providers. J Cancer Res Clin Oncol. 2019;145(2):495–502 Epub 2018/12/13. 10.1007/s00432-018-2813-3 30539283 PMC11810381

[233] Hay CM, Donovan HS, Hartnett EG, et al. Sexual health as part of gynecologic cancer care: what do patients want? Int J Gynecol Cancer. 2018;28(9):1737–1742. 10.1097/IGC.0000000000001376 30358703 PMC7571340

[234] Bedell S, Manders D, Kehoe S, et al. The opinions and practices of providers toward the sexual issues of cervical cancer patients undergoing treatment. Gynecol Oncol. 2017;144(3):586–591. 10.1016/j.ygyno.2016.12.022 28081881

[235] Vermeer WM, Bakker RM, Stiggelbout AM, Creutzberg CL, Kenter GG, ter Kuile MM. Psychosexual support for gynecological cancer survivors: professionals' current practices and need for assistance. Support Care Cancer. 2015;23(3):831–839. 10.1007/s00520-014-2433-7 25218609

[236] Dai Y, Cook OY, Yeganeh L, Huang C, Ding J, Johnson CE. Patient-reported barriers and facilitators to seeking and accessing support in gynecologic and breast cancer survivors with sexual problems: a systematic review of qualitative and quantitative studies. J Sex Med. 2020;17(7):1326–1358. 10.1016/j.jsxm.2020.03.004 32331967

[237] Albers LF, Palacios LAG, Pelger RCM, Elzevier HW. Can the provision of sexual healthcare for oncology patients be improved? A literature review of educational interventions for healthcare professionals. J Cancer Surviv. 2020;14:858–866. 10.1007/s11764-020-00898-4 32488631 PMC7572328

[238] Bingham SL, Semple CJ, Flannagan C, Dunwoody L. Enhancing healthcare professional-led sexual support in cancer care: acceptability and usability of an eLearning resource and its impact on attitudes towards providing sexual support. Psychooncology. 2022;31(9):1555–1563. 10.1002/pon.5993 35781720

[239] McCaughan EM, Flannagan C, Parahoo K, et al. Effects of a brief E-learning resource on sexual attitudes and beliefs of healthcare professionals working in prostate cancer care: a pilot study. Int J Environ Res Public Health. 2021;18(19):10045. 10.3390/ijerph181910045 PubMed PMID: 34639350 34639350 PMC8508566

[240] Reese JB, Lepore SJ, Daly MB, et al. A brief intervention to enhance breast cancer clinicians' communication about sexual health: feasibility, acceptability, and preliminary outcomes. Psychooncology. 2019;28(4):872–879. 10.1002/pon.5036 30811732 PMC6445732

[241] Reese JB, Zimmaro LA, Bober SL, et al. Mobile Technology-Based (mLearning) Intervention to Enhance Breast Cancer Clinicians' Communication About Sexual Health: A Pilot Trial. J Natl Compr Canc Netw 2021;19(10):1133–1140. 10.6004/jnccn.2021.7032 PMC8840991 34388731

[242] Reese JB, Sorice KA, Pollard W, et al. Efficacy of a multimedia intervention in facilitating breast cancer patients' clinical communication about sexual health: results of a randomized controlled trial. Psychooncology. 2020;30:681–690. 10.1002/pon.5613 PMC8113064 33305520

[243] Reese JB, Bober SL, Sorice KA, et al. Starting the conversation: randomized pilot trial of an intervention to promote effective clinical communication about sexual health for gynecologic cancer survivors. J Cancer Surviv. 2024;18:800–809. 10.1007/s11764-022-01327-4 PMC10323044 36604391

[244] Febrina F, Triyoga IF, White M, Marino JL, Peate M. Efficacy of interventions to manage sexual dysfunction in women with cancer: a systematic review. Menopause. 2022;29(5):609–626. https://link.springer.com/article/10.1007/s11764-022-01327-4 35486951 10.1097/GME.0000000000001953

[245] Brennen R, Lin KY, Denehy L, Frawley HC. The effect of pelvic floor muscle interventions on pelvic floor dysfunction after Gynecological cancer treatment: a systematic review. Phys Ther. 2020;100(8):1357–1371. 10.1093/ptj/pzaa081 32367126

[246] Cyr MP, Dostie R, Camden C, et al. Improvements following multimodal pelvic floor physical therapy in gynecological cancer survivors suffering from pain during sexual intercourse: results from a one-year follow-up mixed-method study. PLoS ONE. 2022;17(1):e0262844. 10.1371/journal.pone.0262844 35077479 PMC8789131

[247] Cerentini TM, Schlottgen J, Viana da Rosa P, et al. Clinical and psychological outcomes of the use of vaginal dilators after gynaecological brachytherapy: a randomized clinical trial. Adv Ther. 2019;36(8):1936–1949. 10.1007/s12325-019-01006-4 31209699 PMC6822871

[248] Lubotzky FP, Butow P, Hunt C, et al. A psychosexual rehabilitation booklet increases vaginal dilator adherence and knowledge in women undergoing pelvic radiation therapy for gynaecological or anorectal cancer: a randomised controlled trial. Clin Oncol (R Coll Radiol). 2019;31(2):124–131. 10.1016/j.clon.2018.11.035 30580905

[249] Brennen R, Soh SE, Denehy L, et al. Pelvic floor muscle training delivered via telehealth to treat urinary and/or faecal incontinence after gynaecological cancer surgery: a single cohort feasibility study. Support Care Cancer. 2023;31(10):589. 10.1007/s00520-023-08050-5 37740820 PMC10517895

[250] Bakker RM, Mens JW, de Groot HE, et al. A nurse-led sexual rehabilitation intervention after radiotherapy for gynecological cancer. Support Care Cancer. 2017;25(3):729–737. 10.1007/s00520-016-3453-2 27787681 PMC5266770

[251] Barton DL, Sloan JA, Shuster LT, et al. Impact of vaginal dehydroepiandosterone (DHEA) on vaginal symptoms in female cancer survivors: trial N10C1 (alliance). J Clin Oncol. 2014;32(15_suppl):9507. 10.1200/jco.2014.32.15_suppl.9507

[252] Barton DL, Pugh SL, Ganz PA, et al. Randomized controlled phase II evaluation of two dose levels of bupropion versus placebo for sexual desire in female cancer survivors: NRG-CC004. J Clin Oncol. 2022;40(4):324–334. 10.1200/jco.21.01473 34882500 PMC8797544

[253] Simon J, Portman D, Mabey RG Jr. Long-term safety of ospemifene (52-week extension) in the treatment of vulvar and vaginal atrophy in hysterectomized postmenopausal women. Maturitas. 2014;77(3):274–281. 10.1016/j.maturitas.2013.12.005 24411556

[254] Goldstein SR, Bachmann GA, Koninckx PR, Lin VH, Portman DJ, Ylikorkala O. Ospemifene 12-month safety and efficacy in postmenopausal women with vulvar and vaginal atrophy. Climacteric. 2014;17(2):173–182. 10.3109/13697137.2013.834493 23984673 PMC3971738

[255] De Rosa N, Lavitola G, Giampaolino P, Morra I, Nappi C, Bifulco G. Impact of ospemifene on quality of life and sexual function in young survivors of cervical cancer: a prospective study. Biomed Res Int. 2017;2017:7513610. 10.1155/2017/7513610 28781968 PMC5525090

[256] D'Oria O, Giannini A, Buzzaccarini G, et al. Fractional Co2 laser for vulvo-vaginal atrophy in gynecologic cancer patients: a valid therapeutic choice? A systematic review. Eur J Obstet Gynecol Reprod Biol. 2022;277:84–89. 10.1016/j.ejogrb.2022.08.012 36037664

[257] Angioli R, Stefano S, Filippini M, et al. Effectiveness of CO (2) laser on urogenital syndrome in women with a previous gynecological neoplasia: a multicentric study. Int J Gynecol Cancer. 2020;30(5):590–595. 10.1136/ijgc-2019-001028 32221022

[258] Ma’rifah AR, Afiyanti Y, Huda MH, Chipojola R, Putri YR, Nasution MAT. Effectiveness of psychoeducation intervention among women with gynecological cancer: a systematic review and meta-analysis of randomized controlled trials. Support Care Cancer. 2022;30(10):8271–8285. 10.1007/s00520-022-07277-y 35821448

[259] Schofield P, Gough K, Pascoe M, et al. A nurse- and peer-led psycho-educational intervention to support women with gynaecological cancers receiving curative radiotherapy: the PeNTAGOn randomised controlled trial—ANZGOG 1102. Gynecol Oncol. 2020;159(3):785–793. 10.1016/j.ygyno.2020.09.016 32962898

[260] Brotto LA, Dunkley CR, Breckon E, et al. Integrating quantitative and qualitative methods to evaluate an online psychoeducational program for sexual difficulties in colorectal and gynecologic cancer survivors. J Sex Marital Ther. 2017;43(7):645–662. 10.1080/0092623x.2016.1230805 27592509

[261] Bober SL, Fine E, Recklitis CJ. Sexual health and rehabilitation after ovarian suppression treatment (SHARE-OS): a clinical intervention for young breast cancer survivors. J Cancer Surviv. 2020;14(1):26–30. 10.1007/s11764-019-00800-x 31482477

[262] Schover LR, Strollo S, Stein K, Fallon E, Smith T. Effectiveness trial of an online self-help intervention for sexual problems after cancer. J Sex Marital Ther. 2020;46(6):576–588. 10.1080/0092623X.2020.1762813 32400321

[263] Lin KY, Frawley HC, Denehy L, Feil D, Granger CL. Exercise interventions for patients with gynaecological cancer: a systematic review and meta-analysis. Physiotherapy. 2016;102(4):309–319. 10.1016/j.physio.2016.02.006 27553642

[264] Armbruster SD, Song J, Bradford A, Carmack CL, Lu KH, Basen-Engquist KM. Sexual health of endometrial cancer survivors before and after a physical activity intervention: a retrospective cohort analysis. Gynecol Oncol. 2016;143(3):589–595. 10.1016/j.ygyno.2016.09.016 27678296 PMC5116408

[265] Sung H, Ferlay J, Siegel RL, et al. Global Cancer Statistics 2020: GLOBOCAN estimates of incidence and mortality worldwide for 36 cancers in 185 countries. CA Cancer J Clin. 2021;71(3):209–249. 10.3322/caac.21660 33538338

[266] Clements MB, Beech BB, Atkinson TM, et al. Health-related quality of life after robotic-assisted vs open radical cystectomy: analysis of a randomized trial. J Urol. 2023;209(5):901–910. 10.1097/ju.0000000000003201 36724053 PMC10150857

[267] Mastroianni R, Tuderti G, Anceschi U, et al. Comparison of patient-reported health-related quality of life between open radical cystectomy and robot-assisted radical cystectomy with intracorporeal urinary diversion: interim analysis of a randomised controlled trial. Eur Urol Focus. 2022;8(2):465–471. 10.1016/j.euf.2021.03.002 33712389

[268] Mastroianni R, Tuderti G, Ferriero M, et al. Open vs robotic intracorporeal Padua ileal bladder: functional outcomes of a single-centre RCT. World J Urol. 2023;41(3):739–746. 10.1007/s00345-023-04312-3 36847812

[269] Wilson TG, Guru K, Rosen RC, et al. Best practices in robot-assisted radical cystectomy and urinary reconstruction: recommendations of the Pasadena consensus panel. Eur Urol. 2015;67(3):363–375. 10.1016/j.eururo.2014.12.009 25582930

[270] Xiong X, Qiu S, Yi X, et al. Effect of neurovascular bundle sparing radical cystectomy on post-operative continence and sexual function: a systematic review and meta-analysis. Andrology. 2021;9(1):221–232. 10.1111/andr.12898 32875711

[271] Cassidy RJ, Yang X, Liu T, Thomas M, Nour SG, Jani AB. Neurovascular bundle-sparing radiotherapy for prostate cancer using MRI-CT registration: a dosimetric feasibility study. Med Dosim Winter. 2016;41(4):339–343. 10.1016/j.meddos.2016.08.003 27745996

[272] Ziouziou I, Irani J, Wei JT, et al. Ileal conduit vs orthotopic neobladder: which one offers the best health-related quality of life in patients undergoing radical cystectomy? A systematic review of literature and meta-analysis. Prog Urol. 2018;28(5):241–250. 10.1016/j.purol.2018.02.001 29571902

[273] Chen PY, Chiang PH. Comparisons of quality of life and functional and oncological outcomes after orthotopic neobladder reconstruction: prostate-sparing cystectomy versus conventional radical cystoprostatectomy. Biomed Res Int. 2017;2017:1983428. 10.1155/2017/1983428 28589133 PMC5447259

[274] Jacobs BL, Daignault S, Lee CT, et al. Prostate capsule sparing versus nerve sparing radical cystectomy for bladder cancer: results of a randomized, controlled trial. J Urol. 2015;193(1):64–70. 10.1016/j.juro.2014.07.090 25066875 PMC4368062

[275] Hernández V, Espinos EL, Dunn J, et al. Oncological and functional outcomes of sexual function-preserving cystectomy compared with standard radical cystectomy in men: a systematic review. Urol Oncol. 2017;35(9):539.e17–539.e29. 10.1016/j.urolonc.2017.04.013 28495555

[276] Veskimäe E, Neuzillet Y, Rouanne M, et al. Systematic review of the oncological and functional outcomes of pelvic organ-preserving radical cystectomy (RC) compared with standard RC in women who undergo curative surgery and orthotopic neobladder substitution for bladder cancer. BJU Int. 2017;120(1):12–24. 10.1111/bju.13819 28220653

[277] Volders JH, Haloua MH, Krekel NM, et al. Intraoperative ultrasound guidance in breast-conserving surgery shows superiority in oncological outcome, long-term cosmetic and patient-reported outcomes: final outcomes of a randomized controlled trial (COBALT). Eur J Surg Oncol. 2017;43(4):649–657. 10.1016/j.ejso.2016.11.004 27916314

[278] Toesca A, Sangalli C, Maisonneuve P, et al. A randomized trial of robotic mastectomy versus open surgery in women with breast cancer or BrCA mutation. Ann Surg. 2022;276(1):11–19. 10.1097/sla.0000000000004969 34597010

[279] Wei M, Wu Q, Fan C, et al. Lateral pelvic lymph node dissection after neoadjuvant chemo-radiation for preoperative enlarged lateral nodes in advanced low rectal cancer: study protocol for a randomized controlled trial. Trials. 2016;17:561. 10.1186/s13063-016-1695-4 PMC5123316 27884204

[280] Bogani G, Rossetti DO, Ditto A, et al. Nerve-sparing approach improves outcomes of patients undergoing minimally invasive radical hysterectomy: a systematic review and meta-analysis. J Minim Invasive Gynecol. 2018;25(3):402–410. 10.1016/j.jmig.2017.11.014 29191471

[281] Hasenburg A, Sehouli J, Lampe B, et al. LION-PAW (lymphadenectomy in ovarian neoplasm) sexual function assessment: a prospective sub-study of the LION trial. Int J Gynecol Cancer. 2020;30:1548–1553. 10.1136/ijgc-2020-001551 32938723

[282] Bekelman JE, Rumble RB, Chen RC, et al. Clinically localized prostate cancer: ASCO Clinical Practice Guideline Endorsement of an American Urological Association/American Society for Radiation Oncology/Society of Urologic Oncology Guideline. J Clin Oncol. 2018;36(32):3251–3258. 10.1200/JCO.18.00606 30183466

[283] Ilic D, Evans SM, Allan CA, Jung JH, Murphy D, Frydenberg M. Laparoscopic and robotic-assisted versus open radical prostatectomy for the treatment of localised prostate cancer. Cochrane Database Syst Rev. 2017;9:CD009625. 10.1002/14651858.CD009625.pub2 28895658 PMC6486168

[284] Lantz A, Bock D, Akre O, et al. Functional and oncological outcomes after open versus robot-assisted laparoscopic radical prostatectomy for localised prostate cancer: 8-year follow-up. Eur Urol. 2021;80(5):650–660. 10.1016/j.eururo.2021.07.025 34538508

[285] Yaxley JW, Coughlin GD, Chambers SK, et al. Robot-assisted laparoscopic prostatectomy versus open radical retropubic prostatectomy: early outcomes from a randomised controlled phase 3 study. Lancet. 2016;388(10049):1057–1066. 10.1016/s0140-6736(16)30592-x 27474375

[286] Holze S, Lemaire E, Mende M, et al. Quality of life after robotic-assisted and laparoscopic radical prostatectomy: results of a multicenter randomized controlled trial (LAP-01). Prostate. 2022;82(8):894–903. 10.1002/pros.24332 35254665

[287] Coughlin GD, Yaxley JW, Chambers SK, et al. Robot-assisted laparoscopic prostatectomy versus open radical retropubic prostatectomy: 24-month outcomes from a randomised controlled study. Lancet Oncol. 2018;19(8):1051–1060. 10.1016/s1470-2045(18)30357-7 30017351

[288] Gilbert SM, Dunn RL, Miller DC, et al. Functional outcomes following nerve sparing prostatectomy augmented with seminal vesicle sparing compared to standard nerve sparing prostatectomy: results from a randomized controlled trial. J Urol. 2017;198(3):600–607. 10.1016/j.juro.2017.03.133 28392393

[289] Feng T, Heulitt G, Lee JJ, Liao M, Li HF, Porter JR. Randomised comparison of techniques for control of the dorsal venous complex during robot-assisted laparoscopic radical prostatectomy. BJU Int. 2020;126(5):586–594. 10.1111/bju.15133 32521115

[290] Siltari A, Riikonen J, Murtola TJ. Preservation of endopelvic fascia: effects on postoperative incontinence and sexual function—a randomized clinical trial. J Sex Med. 2021;18(2):327–338. 10.1016/j.jsxm.2020.11.003 33358241

[291] Hatiboglu G, Simpfendörfer T, Uhlmann L, et al. A prospective randomized controlled trial for assessment of perineal hydrodissection technique for nervesparing robot assisted radical prostatectomy. Int J Med Robot. 2017;13(4):e1835. 10.1002/rcs.1835 28544071

[292] Menon M, Dalela D, Jamil M, et al. Functional recovery, oncologic outcomes and postoperative complications after robot-assisted radical prostatectomy: an evidence-based analysis comparing the Retzius sparing and standard approaches. J Urol. 2018;199(5):1210–1217. 10.1016/j.juro.2017.11.115 29225060

[293] Nguyen LN, Head L, Witiuk K, et al. The risks and benefits of cavernous neurovascular bundle sparing during radical prostatectomy: a systematic review and meta-analysis. J Urol. 2017;198(4):760–769. 10.1016/j.juro.2017.02.3344 28286069

[294] Sibert NT, Pfaff H, Breidenbach C, et al. Variation across operating sites in urinary and sexual outcomes after radical prostatectomy in localized and locally advanced prostate cancer. World J Urol. 2022;40(6):1437–1446. 10.1007/s00345-022-03985-6 35347412

[295] Rosenberg JE, Jung JH, Edgerton Z, et al. Retzius-sparing versus standard robotic-assisted laparoscopic prostatectomy for the treatment of clinically localized prostate cancer. Cochrane Database Syst Rev. 2020;8:CD013641. 10.1002/14651858.CD013641.pub2 32813279 PMC7437391

[296] Krieger L, Holze S, Mende M, et al. Extended pelvic lymph node dissection does not affect functional outcomes during bilateral nerve-sparing radical prostatectomy. Urol Int. 2022;106(11):1136–1144. 10.1159/000526113 36096125

[297] Mercieca-Bebber R, Eggins R, Brown K, et al. Patient-reported bowel, urinary, and sexual outcomes after laparoscopic-assisted resection or open resection for rectal cancer: the Australasian Laparoscopic Cancer of the Rectum Randomized Clinical Trial (ALaCart). Ann Surg. 2023;277(3):449–455. 10.1097/sla.0000000000005412 35166265

[298] Kim MJ, Park SC, Park JW, et al. Robot-assisted versus laparoscopic surgery for rectal cancer: a phase II open label prospective randomized controlled trial. Ann Surg. 2018;267(2):243–251. 10.1097/sla.0000000000002321 28549014

[299] Galata C, Vassilev G, Haas F, et al. Clinical, oncological, and functional outcomes of Da Vinci (Xi)-assisted versus conventional laparoscopic resection for rectal cancer: a prospective, controlled cohort study of 51 consecutive cases. Int J Color Dis. 2019;34:1907–1914. 10.1007/s00384-019-03397-w 31642968

[300] Feng Q, Tang W, Zhang Z, et al. Robotic versus laparoscopic abdominoperineal resections for low rectal cancer: a single-center randomized controlled trial. J Surg Oncol. 2022;126(8):1481–1493. 10.1002/jso.27076 36036889

[301] Jayne D, Pigazzi A, Marshall H, et al. Effect of robotic-assisted vs conventional laparoscopic surgery on risk of conversion to open laparotomy among patients undergoing resection for rectal cancer: the ROLARR randomized clinical trial. JAMA. 2017;318(16):1569–1580. 10.1001/jama.2017.7219 29067426 PMC5818805

[302] Wei B, Zheng Z, Fang J, et al. Effect of Denonvilliers' fascia preservation versus resection during laparoscopic total mesorectal excision on postoperative urogenital function of male rectal cancer patients: initial results of Chinese PUF-01 randomized clinical trial. Ann Surg. 2021;274(6):e473–e480. 10.1097/sla.0000000000004591 33234798

[303] Liu J, Huang P, Liang Q, Yang X, Zheng Z, Wei H. Preservation of Denonvilliers' fascia for nerve-sparing laparoscopic total mesorectal excision: a neuro-histological study. Clin Anat. 2019;32(3):439–445. 10.1002/ca.23336 30664277

[304] Pontallier A, Denost Q, Van Geluwe B, Adam JP, Celerier B, Rullier E. Potential sexual function improvement by using transanal mesorectal approach for laparoscopic low rectal cancer excision. Surg Endosc. 2016;30:4924–4933. 10.1007/s00464-016-4833-x 26944728

[305] Pallisera-Lloveras A, Planelles-Soler P, Hannaoui N, et al. Dissection of the inferior mesenteric vein versus of the inferior mesenteric artery for the genitourinary function after laparoscopic approach of rectal cancer surgery: a randomized controlled trial. BMC Urol. 2019;19:75. 10.1186/s12894-019-0501-5 PMC6683580 31382934

[306] Mari GM, Crippa J, Cocozza E, et al. Low ligation of inferior mesenteric artery in laparoscopic anterior resection for rectal cancer reduces genitourinary dysfunction: results from a randomized controlled trial (HIGHLOW trial). Ann Surg. 2019;269(6):1018–1024. 10.1097/sla.0000000000002947 31082897

[307] Feng B, Lu J, Zhang S, et al. Laparoscopic abdominoperineal excision with trans-abdominal individualized levator transection: interim analysis of a randomized controlled trial. Color Dis. 2017;19(7):O246–o252. 10.1111/codi.13711 28477432

[308] Kachnic LA, Winter KA, Myerson RJ, et al. Long-term outcomes of NRG oncology/RTOG 0529: a phase 2 evaluation of dose-painted intensity modulated radiation therapy in combination with 5-fluorouracil and mitomycin-C for the reduction of acute morbidity in anal canal cancer. Int J Radiat Oncol Biol Phys. 2022;112(1):146–157. 10.1016/j.ijrobp.2021.08.008 34400269 PMC8688291

[309] Joseph K, Balushi MA, Ghosh S, et al. Long-term patient-reported quality of life of anal cancer survivors treated with intensity modulated radiation therapy and concurrent chemotherapy: results from a prospective phase II trial. Int J Radiat Oncol Biol Phys. 2023;117(2):434–445. 10.1016/j.ijrobp.2023.04.023 37148982

[310] McLachlan SA, Fisher RJ, Zalcberg J, et al. The impact on health-related quality of life in the first 12 months: a randomised comparison of preoperative short-course radiation versus long-course chemoradiation for T3 rectal cancer (trans-Tasman radiation oncology group trial 01.04). Eur J Cancer. 2016;55:15–26. 10.1016/j.ejca.2015.10.060 26771873

[311] Marshall DC, Ghiassi-Nejad Z, Powers A, et al. A first radiotherapy application of functional bulboclitoris anatomy, a novel female sexual organ-at-risk, and organ-sparing feasibility study. Br J Radiol. 2021;94:20201139. 10.1259/bjr.20201139 PMC8764912 34192475

[312] Marshall DC, Tarras ES, Ali A, Bloom J, Torres MA, Kahn JM. Female erectile tissues and sexual dysfunction after pelvic radiotherapy: a scoping review. CA Cancer J Clin. 2022;72(4):353–359. 10.3322/caac.21726 35298025 PMC9262811

[313] Briere TM, Crane CH, Beddar S, et al. Reproducibility and genital sparing with a vaginal dilator used for female anal cancer patients. Radiother Oncol. 2012;104(2):161–166. 10.1016/j.radonc.2012.05.011 22841019

[314] Westerveld H, Kirchheiner K, Nout RA, et al. Dose-effect relationship between vaginal dose points and vaginal stenosis in cervical cancer: an EMBRACE-I sub-study. Radiother Oncol. 2022;168:8–15. 10.1016/j.radonc.2021.12.034 35063582

[315] Kirchheiner K, Nout RA, Lindegaard JC, et al. Dose-effect relationship and risk factors for vaginal stenosis after definitive radio(chemo)therapy with image-guided brachytherapy for locally advanced cervical cancer in the EMBRACE study. Radiother Oncol. 2016;118(1):160–166. 10.1016/j.radonc.2015.12.025 26780997

[316] de Boer SM, Nout RA, Jürgenliemk-Schulz IM, et al. Long-term impact of endometrial cancer diagnosis and treatment on health-related quality of life and cancer survivorship: results from the randomized PORTEC-2 trial. Int J Radiat Oncol Biol Phys. 2015;93(4):797–809. 10.1016/j.ijrobp.2015.08.023 26530748

[317] Escande A, Haie-Meder C, Mazeron R, et al. Brachytherapy for conservative treatment of invasive penile carcinoma: prognostic factors and long-term analysis of outcome. Int J Radiat Oncol Biol Phys. 2017;99(3):563–570. 10.1016/j.ijrobp.2017.02.090 28501419

[318] Cordoba A, Escande A, Lopez S, et al. Low-dose brachytherapy for early stage penile cancer: a 20-year single-institution study (73 patients). Radiat Oncol. 2016;11:96. 10.1186/s13014-016-0676-9 27464910 PMC4964092

[319] Seibold J, Strnad V, Fietkau R. Organ-sparing treatment of penile cancer with interstitial pulsed-dose-rate brachytherapy. Strahlenther Onkol. 2016;192(7):467–472. 10.1007/s00066-016-0968-x 27276876

[320] Cloitre M, Valerio M, Mampuya A, et al. Toxicity, quality of life, and PSA control after 50 Gy stereotactic body radiation therapy to the dominant intraprostatic nodule with the use of a rectal spacer: results of a phase I/II study. Br J Radiol. 2023;96(1145):20220803. 10.1259/bjr.20220803 36745031 PMC10161910

[321] Wortel RC, Oomen-de Hoop E, Heemsbergen WD, Pos FJ, Incrocci L. Moderate hypofractionation in intermediate- and high-risk, localized prostate cancer: health-related quality of life from the randomized, phase 3 HYPRO trial. Int J Radiat Oncol Biol Phys. 2019;103(4):823–833. 10.1016/j.ijrobp.2018.11.020 30458234

[322] Wortel RC, Pos FJ, Heemsbergen WD, Incrocci L. Sexual function after hypofractionated versus conventionally fractionated radiotherapy for prostate cancer: results from the randomized phase III HYPRO trial. J Sex Med. 2016;13:1695–1703. 10.1016/j.jsxm.2016.08.012 27665195

[323] Vargas CE, Hartsell WF, Dunn M, et al. Hypofractionated versus standard fractionated proton-beam therapy for low-risk prostate cancer: interim results of a randomized trial PCG GU 002. Am J Clin Oncol. 2018;41(2):115–120. 10.1097/coc.0000000000000241 26523442

[324] Staffurth JN, Haviland JS, Wilkins A, et al. Impact of hypofractionated radiotherapy on patient-reported outcomes in prostate cancer: results up to 5 yr in the CHHiP trial (CRUK/06/016). Eur Urol Oncol. 2021;4(6):980–992. 10.1016/j.euo.2021.07.005 34489210 PMC8674146

[325] Bruner DW, Pugh SL, Lee WR, et al. Quality of life in patients with low-risk prostate cancer treated with hypofractionated vs conventional radiotherapy: a phase 3 randomized clinical trial. JAMA Oncol. 2019;5(5):664–670. 10.1001/jamaoncol.2018.6752 30763425 PMC6459051

[326] Jackson WC, Dess RT, Litzenberg DW, et al. A multi-institutional phase 2 trial of prostate stereotactic body radiation therapy (SBRT) using continuous real-time evaluation of prostate motion with patient-reported quality of life. Pract Radiat Oncol. 2018;8(1):40–47. 10.1016/j.prro.2017.08.004 29304991

[327] Zilli T, Jorcano S, Bral S, et al. Once-a-week or every-other-day urethra-sparing prostate cancer stereotactic body radiotherapy, a randomized phase II trial: 18 months follow-up results. Cancer Med. 2020;9(9):3097–3106. 10.1002/cam4.2966 32160416 PMC7196054

[328] Johnson SB, Soulos PR, Shafman TD, et al. Patient-reported quality of life after stereotactic body radiation therapy versus moderate hypofractionation for clinically localized prostate cancer. Radiother Oncol. 2016;121(2):294–298. 10.1016/j.radonc.2016.10.013 27890426

[329] Fuller DB, Falchook AD, Crabtree T, et al. Phase 2 Multicenter trial of heterogeneous-dosing stereotactic body radiotherapy for low- and intermediate-risk prostate cancer: 5-year outcomes. Eur Urol Oncol. 2018;1(6):540–547. 10.1016/j.euo.2018.06.013 31158102

[330] Fransson P, Nilsson P, Gunnlaugsson A, et al. Ultra-hypofractionated versus conventionally fractionated radiotherapy for prostate cancer (HYPO-RT-PC): patient-reported quality-of-life outcomes of a randomised, controlled, non-inferiority, phase 3 trial. Lancet Oncol. 2021;22(2):235–245. 10.1016/s1470-2045(20)30581-7 33444529

[331] Boyer MJ, Papagikos MA, Kiteley R, Vujaskovic Z, Wu J, Lee WR. Toxicity and quality of life report of a phase II study of stereotactic body radiotherapy (SBRT) for low and intermediate risk prostate cancer. Radiat Oncol. 2017;12(1):14. 10.1186/s13014-016-0758-8 28086825 PMC5237336

[332] Zapatero A, Roch M, Castro Tejero P, et al. MRI-guided focal boost to dominant intraprostatic lesion using volumetric modulated arc therapy in prostate cancer. Results of a phase II trial. Br J Radiol. 2022;95(1131):20210683. 10.1259/bjr.20210683 34538073 PMC8978233

[333] Pasquier D, Peiffert D, Nickers P, et al. A multicenter phase 2 study of hypofractionated stereostatic boost in intermediate risk prostate carcinoma: a 5-year analysis of the CKNO-PRO trial. Int J Radiat Oncol Biol Phys. 2020;106(1):116–123. 10.1016/j.ijrobp.2019.09.039 31604131

[334] Pasquier D, Nickers P, Peiffert D, et al. Hypofractionated stereotactic boost in intermediate risk prostate carcinoma: preliminary results of a multicenter phase II trial (CKNO-PRO). PLoS ONE. 2017;12(11):e0187794. 10.1371/journal.pone.0187794 29190707 PMC5708754

[335] Bryant C, Smith TL, Henderson RH, et al. Five-year biochemical results, toxicity, and patient-reported quality of life after delivery of dose-escalated image guided proton therapy for prostate cancer. Int J Radiat Oncol Biol Phys. 2016;95(1):422–434. 10.1016/j.ijrobp.2016.02.038 27084658

[336] Bruner DW, Hunt D, Michalski JM, et al. Preliminary patient-reported outcomes analysis of 3-dimensional radiation therapy versus intensity-modulated radiation therapy on the high-dose arm of the Radiation Therapy Oncology Group (RTOG) 0126 prostate cancer trial. Cancer. 2015;121(14):2422–2430. 10.1002/cncr.29362 25847819 PMC4490066

[337] Morton G, Chung HT, McGuffin M, et al. Prostate high dose-rate brachytherapy as monotherapy for low and intermediate risk prostate cancer: early toxicity and quality-of life results from a randomized phase II clinical trial of one fraction of 19Gy or two fractions of 13.5Gy. Radiother Oncol. 2017;122(1):87–92. 10.1016/j.radonc.2016.10.019 27823821

[338] Morris BA, Holmes EE, Anger NJ, et al. Toxicity and patient-reported quality-of-life outcomes after prostate stereotactic body radiation therapy with focal boost to magnetic resonance imaging-identified prostate cancer lesions: results of a phase 2 trial. Int J Radiat Oncol Biol Phys. 2023;117(3):613–623. 10.1016/j.ijrobp.2023.05.004 37179035

[339] Marvaso G, Gugliandolo SG, Bellerba F, et al. Phase II prospective trial "give me five" short-term high precision radiotherapy for early prostate cancer with simultaneous boost to the dominant intraprostatic lesion: the impact of toxicity on quality of life (AIRC IG-13218). Med Oncol. 2020;37(8):74. 10.1007/s12032-020-01397-3 32725443

[340] Lukka HR, Pugh SL, Bruner DW, et al. Patient reported outcomes in NRG oncology RTOG 0938, evaluating two ultrahypofractionated regimens for prostate cancer. Int J Radiat Oncol Biol Phys. 2018;102(2):287–295. 10.1016/j.ijrobp.2018.06.008 29913254 PMC6248906

[341] Ong WL, Cheung P, Chung H, et al. Two-fraction stereotactic ablative radiotherapy with simultaneous boost to MRI-defined dominant intra-prostatic lesion—results from the 2SMART phase 2 trial. Radiother Oncol. 2023;181:109503. 10.1016/j.radonc.2023.109503 36754232

[342] Yang F, Ghosh S, Yee D, et al. Conventional versus hypofractionated radiation for high-risk prostate cancer patients (CHIRP): 24-month patient-reported outcomes of the randomized phase 2 CHIRP trial. Int J Radiat Oncol Biol Phys. 2022;114(1):99–107. 10.1016/j.ijrobp.2022.04.045 35537578

[343] Sanmamed N, Locke G, Crook J, et al. Long-term biochemical control of a prospective cohort of prostate cancer patients treated with interstitial brachytherapy versus radical prostatectomy. Clin Oncol (R Coll Radiol). 2023;35(4):262–268. 10.1016/j.clon.2023.01.006 36737311

[344] Hamstra DA, Mariados N, Sylvester J, et al. Sexual quality of life following prostate intensity modulated radiation therapy (IMRT) with a rectal/prostate spacer: secondary analysis of a phase 3 trial. Pract Radiat Oncol. 2018;8(1):e7–e15. 10.1016/j.prro.2017.07.008 28951089

[345] Yang Q, Xia L, Lin M, et al. The impact of induction chemotherapy on long-term quality of life in patients with locoregionally advanced nasopharyngeal carcinoma: outcomes from a randomised phase 3 trial. Oral Oncol. 2021;121:105494. 10.1016/j.oraloncology.2021.105494 34425533

[346] Dijkstra EA, Hospers GAP, Kranenbarg EM, et al. Quality of life and late toxicity after short-course radiotherapy followed by chemotherapy or chemoradiotherapy for locally advanced rectal cancer—the RAPIDO trial. Radiother Oncol. 2022;171:69–76. 10.1016/j.radonc.2022.04.013 35447283

[347] Conroy T, Bosset JF, Etienne PL, et al. Neoadjuvant chemotherapy with FOLFIRINOX and preoperative chemoradiotherapy for patients with locally advanced rectal cancer (UNICANCER-PRODIGE 23): a multicentre, randomised, open-label, phase 3 trial. Lancet Oncol. 2021;22(5):702–715. 10.1016/S1470-2045(21)00079-6 33862000

[348] Bascoul-Mollevi C, Gourgou S, Borg C, et al. Neoadjuvant chemotherapy with FOLFIRINOX and preoperative chemoradiotherapy for patients with locally advanced rectal cancer (UNICANCER PRODIGE 23): health-related quality of life longitudinal analysis. Eur J Cancer. 2023;186:151–165. 10.1016/j.ejca.2023.03.021 37068407

[349] Sclafani F, Peckitt C, Cunningham D, et al. Short- and long-term quality of life and bowel function in patients with MRI-defined, high-risk, locally advanced rectal cancer treated with an intensified neoadjuvant strategy in the randomized phase 2 EXPERT-C trial. Int J Radiat Oncol Biol Phys. 2015;93(2):303–312. 10.1016/j.ijrobp.2015.03.038 26031368

[350] Post CCB, de Boer SM, Powell ME, et al. Long-term toxicity and health-related quality of life after adjuvant chemoradiation therapy or radiation therapy alone for high-risk endometrial cancer in the randomized PORTEC-3 trial. Int J Radiat Oncol Biol Phys. 2021;109(4):975–986. 10.1016/j.ijrobp.2020.10.030 33129910

[351] Hall E, Hussain SA, Porta N, et al. Chemoradiotherapy in muscle-invasive bladder cancer: 10-yr follow-up of the phase 3 randomised controlled BC2001 trial. Eur Urol. 2022;82(3):273–279. 10.1016/j.eururo.2022.04.017 35577644

[352] Donovan JL, Hamdy FC, Lane JA, et al. Patient-reported outcomes after monitoring, surgery, or radiotherapy for prostate cancer. N Engl J Med. 2016;375(15):1425–1437. 10.1056/NEJMoa1606221 27626365 PMC5134995

[353] Hamdy FC, Donovan JL, Lane JA, et al. Active monitoring, radical prostatectomy and radical radiotherapy in PSA-detected clinically localised prostate cancer: the ProtecT three-arm RCT. Health Technol Assess. 2020;24(37):1–176. 10.3310/hta24370 PMC7443739 32773013

[354] Lane JA, Donovan JL, Young GJ, et al. Functional and quality of life outcomes of localised prostate cancer treatments (Prostate Testing for Cancer and Treatment [ProtecT] study). BJU Int. 2022;130(3):370–380. 10.1111/bju.15739 35373443 PMC9543725

[355] Neal DE, Metcalfe C, Donovan JL, et al. Ten-year mortality, disease progression, and treatment-related side effects in men with localised prostate cancer from the ProtecT randomised controlled trial according to treatment received. Eur Urol. 2020;77(3):320–330. 10.1016/j.eururo.2019.10.030 31771797

[356] Wilt TJ, Jones KM, Barry MJ, et al. Follow-up of prostatectomy versus observation for early prostate cancer. N Engl J Med. 2017;377(2):132–142. 10.1056/NEJMoa1615869 28700844

[357] Carlsson S, Jäderling F, Wallerstedt A, et al. Oncological and functional outcomes 1 year after radical prostatectomy for very-low-risk prostate cancer: results from the prospective LAPPRO trial. BJU Int. 2016;118(2):205–212. 10.1111/bju.13444 26867018 PMC4947001

[358] Rodda S, Morris WJ, Hamm J, Duncan G. ASCENDE-RT: an analysis of health-related quality of life for a randomized trial comparing low-dose-rate brachytherapy boost with dose-escalated external beam boost for high- and intermediate-risk prostate cancer. Int J Radiat Oncol Biol Phys. 2017;98(3):581–589. 10.1016/j.ijrobp.2017.02.027 28581398

[359] Dickstein DR, Edwards CR, Lehrer EJ, et al. Sexual health and treatment-related sexual dysfunction in sexual and gender minorities with prostate cancer. Nat Rev Urol. 2023;20(6):332–355. 10.1038/s41585-023-00778-3 37217695 PMC10389287

[360] Barry Delongchamps N, Schull A, Anract J, et al. Feasibility and safety of targeted focal microwave ablation of the index tumor in patients with low to intermediate risk prostate cancer: results of the FOSTINE trial. PLoS ONE. 2021;16(7):e0252040. 10.1371/journal.pone.0252040 34260598 PMC8279354

[361] Natarajan S, Raman S, Priester AM, et al. Focal laser ablation of prostate cancer: phase I clinical trial. J Urol. 2016;196(1):68–75. 10.1016/j.juro.2015.12.083 26748164

[362] Tan YG, Law YM, Ngo NT, et al. Patient-reported functional outcomes and oncological control after primary focal cryotherapy for clinically significant prostate cancer: a phase II mandatory biopsy-monitored study. Prostate. 2023;83(8):781–791. 10.1002/pros.24517 36895163 PMC10952298

[363] Gregg JR, Borregales LD, Choi H, et al. Prospective trial of regional (hockey-stick) prostate cryoablation: oncologic and quality of life outcomes. World J Urol. 2021;39(9):3259–3264. 10.1007/s00345-020-03575-4 33454813 PMC9810085

[364] Eggener SE, Yousuf A, Watson S, Wang S, Oto A. Phase II evaluation of magnetic resonance imaging guided focal laser ablation of prostate cancer. J Urol. 2016;196(6):1670–1675. 10.1016/j.juro.2016.07.074 27449263

[365] Taneja SS, Bennett J, Coleman J, et al. Final results of a phase I/II Multicenter trial of WST11 vascular targeted photodynamic therapy for hemi-ablation of the prostate in men with unilateral low risk prostate cancer performed in the United States. J Urol. 2016;196(4):1096–1104. 10.1016/j.juro.2016.05.113 27291652 PMC5483996

[366] Tay KJ, Cheng CWS, Lau WKO, Khoo J, Thng CH, Kwek JW. Focal therapy for prostate cancer with in-bore MR-guided focused ultrasound: two-year follow-up of a phase I trial-complications and functional outcomes. Radiology. 2017;285(2):620–628. 10.1148/radiol.2017161650 28654336

[367] Garcia-Aguilar J, Patil S, Gollub MJ, et al. Organ preservation in patients with rectal adenocarcinoma treated with total neoadjuvant therapy. J Clin Oncol. 2022;40(23):2546–2556. 10.1200/JCO.22.00032 35483010 PMC9362876

[368] Basch E, Dueck AC, Mitchell SA, et al. Patient-reported outcomes during and after treatment for locally advanced rectal cancer in the PROSPECT trial (alliance N1048). J Clin Oncol. 2023;41(21):3724–3734. 10.1200/JCO.23.00903 37270691 PMC10351948

